# A potent subset of *Mycobacterium tuberculosis* glycoproteins as relevant candidates for vaccine and therapeutic target

**DOI:** 10.1038/s41598-023-49665-2

**Published:** 2023-12-14

**Authors:** Shamsi Yari, Parviz Afrough, Fatemeh Yari, Morteza Ghazanfari Jajin, Abolfazl Fateh, Alireza Hadizadeh Tasbiti

**Affiliations:** 1 TB Protein Chemistry Lab, Tuberculosis and Pulmonary Research Department, Pasteur Institute of Iran, Pasteur Ave, Tehran, 13164 Iran; 2 Hepatitis Research Center, Shahid Rahimi Hospital, Aligoudarz School of Nursing, Lorestan University of Medical Science, Khorramabad, Iran; 3 Blood Transfusion Research Center, High Institute for Research and Education in Transfusion, Tehran, Iran

**Keywords:** Vaccines, Glycobiology

## Abstract

Tuberculosis (TB) remains one of the most afflictive bacterial infections globally. In high burden TB countries, surveillance, diagnosis and treatment of drug resistant TB (RR and X/MDRTB) display a crucial public health challenge. Therefore, we need new TB vaccines; diagnostic and therapeutic strategies to briskly prevent disease promotion; reduce drug-resistant TB and protect everyone from disease. The study identified various potent membrane and cell wall *M. tuberculosis* glycolipoproteins that are relevant for diagnostics, drug and vaccine discovery. A *M. tuberculosis* Proskauer and Beck broth culture was extracted for total proteins by ammonium sulfate method. After ConA-Affinity Chromatography reputed glycoproteins were collected followed by 2DE gel electrophoresis and LC Mass spectrometry. A total of 293 glycoproteins were identified using GlycoPP and IEDB database. Probable conserved trans-membrane protein (Rv0954), LpqN (Rv0583), PPE68 (Rv3873), Phosphate-binding protein (Rv0932c), PPE61 (Rv3532) and LprA (Rv1270c), had the highest glycosylation percentage value with 13.86%, 11.84%, 11.68%, 11.1%, 10.59% and10.2%, respectively. Our study discloses several dominant glycoproteins that play roles in *M. tuberculosis* survival, and immunogenicity. These include glycoproteins involved in antigenicity, transport and biosynthesis of *M. tuberculosis* cell envelope, pathogen-host interaction and drug efflux pumps, which are considered as a feasible drug targets or TB new vaccine candidates.

## Introduction

Tuberculosis (TB) remains a warning to global public health and is one of the main causes of death worldwide. Prior to the SARS-CoV-2 pandemic, tuberculosis was the dominant cause of death from a single infectious agent, ranking over AIDS or HIV^1,2^. According to global tuberculosis report in 2022, the estimated number of deaths from tuberculosis, increased between 2019 and 2021 (including 187,000 people with HIV**)** and also the burden of resistant to at least one first line anti TB (DR-TB) is estimated to have enhanced from 2020 to 2021^2^. These reports demonstrate the urgency in perception the pathogenicity of *M. tuberculosis*, as well as the inevitability to promote novel therapeutic strategies for the treatment or prevent the disease^3^. Therefore, contrastive analyses of *M. tuberculosis* isolates have determined pathogenicity and virulence factors required for *M. tuberculosis* survival and distribution inside the host, as well candidate proteins (genes) for the development of new TB vaccines or drugs^4^. Membrane, cell wall and secreted proteins in mycobacteria interact with the host and play crucial roles in pathogenicity. These proteins are investigated as protein diagnostic markers, probable drug targets or vaccine antigen candidates, and more currently distinguished consideration is being given to the function of their post-translational modifications (PTMs)^5^. Post-translational modifications (PTMs) are one of the most important alterations in any protein function. These common PTMs include acetylation, phosphorylation, and glycosylation alter protein regulation, stability, folding, conformation and function^6^. Protein glycosylation is an enzyme-catalyzed post translational modification preserved over all domains of life. There are two types of protein glycosylation that can occur in asparagine residues, N-linked glycosylation, while O-linked glycosylation occurs on the side chain of either serine or threonine residues. In mycobacteria, O-linked glycosylation is catalyzed by O-mannosyltransferases (PMTs) which transfers the first mannose units onto serine or threonine residues of the substrate. Deletion mutants of PMT indicated the significance of protein glycosylation in mycobacterial resistance and virulence. Furthermore, the activity of PMT is related to protein secretion and *Mycobacterium tuberculosis* PMTs demonstrates enhanced sigma factor (SigH) expression as an important factor involved in the mycobacterial response to oxidative stress. Many antimycobacterial resistance genes are glycosylated, including first line drug resistance determinants for instance: *KatG* (Isoniazid), *rpoB* (Rifampin), *ethA* (Ethambutol), *blaC* (ß-lactam), and *gyrB* genes (fluoroquinolone) also several reported antibiotic efflux pump proteins (efflux ATP binding Rv0194, esterase LipC Rv2994 and ATP binding transporter Rv1273c)^7–9^.

Glycosylation in *M. tuberculosis* has been identified in outer exposed proteins and generally found in association with acylation in mycobacterial proteins^8^. Moreover, the particular *M. tuberculosis* glycoconjugates in the cell wall are the prominent basis for host pathogen interactions, antigenicity and virulence^7^ and are therefore, identification of *M. tuberculosis* glycoproteins and their activity is significant both for recognition of pathogenesis and development of new TB vaccines, anti-TB drugs and biomarkers. Thus, in the present study, we employed concanavalin affinity chromatography, two*-*dimensional gel electrophoresis (2DE), mass spectrometry and bioinformatics analysis to identify putative glycoproteins in *M. tuberculosis* membrane and secretory proteins to explore the function of protein glycosylation in *M. tuberculosis* survival, antigenicity and Immunogenicity that are suitable for diagnostics and vaccine discovery.

## Material and methods

### Mycobacterial strains and protein extraction

The protocols and data collections were approved by the Research Ethics Committee, Pasteur Institute of Iran, Tehran (IR.PII.REC.1397.031).

*M. tuberculosis* strain (TB − 1039) was obtained from Tuberculosis culture collection at Pasteur Institute of Iran, Tehran. Bacteria were cultured on 976 Proskauer and Beck broth medium at 37 °C for 4–5 weeks. Bacteria (cells) were elicited from the broth culture by centrifugation at 7000 rpm for 35 min and washed with PBS (phosphate-buffered saline) pH 7.4. The aggregated mycobacterial cells were suspended in cell lysis buffer contains 1 mM PMSF (protease inhibitor), 20 mM EDTA, 10% Glycerol, 40 mM Tris, 0.5% Triton X114, 1 μg/ml DNase and 0.02% sodium azide then the bacilli sonicated for 35 min at 60 HZ on ice and thereafter centrifuged at 6000 rpm for 35 min at − 5 °C. Finally, extraction of cytoplasmic proteins were done by ammonium sulfate and dialyzed against PBS, pH 7.4^10^. Regarding, secretory protein purification, the supernatant was collected and protein precipitation was performed by saturated ammonium sulfate, 0.5 g/mL (NH_4_)_2_SO_4_ at 4 °C and dialyzed against PBS pH 7.3, under similar conditions. Both cytoplasmic and secretory protein contents were quantified by Bradford’s method^11^.

### ConA-lectin affinity chromatography

ConA-affinity chromatography was performed in a chromatographic column (25 × 1.7 cm) with the use of Concanavalin A Sepharose 4B (Sigma). As a first step, 2 mL ConA column was equilibrated with washing buffer contains 5 mM CaCl_2_, 1 M NaCl, 5 mM MnCl_2_ and 5 mM MgCl_2_ pH 6.5. The samples (protein contents) were incubated in the binding/Equilibration buffer (20 mM Tris–HCL, 0.5 M NaCl, 5 mM MnCl_2_, 5 mM MgCl_2_ and 5 mM CaCl_2_, pH 6.5) for 1 h and then the column was loaded with 2.5 mg/ml of protein contents followed by several washes with cationic buffer. Gradient elution mode is employed for elution of chromatographic peaks therefore 0.2, 0.5, 0.8 and 1 M Methyl-α-d mannopyranoside (Sigma) were used for elution. Enriched fractions of reputed glycoproteins were collected and their UV absorbance measured at 280 nm^12,13^.

### One*-*dimensional gel electrophoresis

One-dimensional gel electrophoresis, SDS-PAGE of ConA purified protein was carried out for vertical electrophoresis (PROTEAN Tetra Cell, Bio-Rad) based on Laemmli method^14^.

### Two-dimensional gel electrophoresis (2DE)

Two*-*dimensional gel electrophoresis, 2DE*-*PAGE, was performed by the Ettan IPGphor 3 apparatus (GE HealthCare). Passive rehydration in the IEF tray utilized to isoelectric focusing step. Isoelectric focusing (IEF) buffer consist of 0.3% carrier ampholyte, 100 mM DTT (Dithiothreitol), 5 M urea, 2 mM TBP (Tributylphosphine), 5% CHAPS, 50 mM Tris HCl pH 7.4, and 1.5 M Thiourea. Four hundred μL of the protein contents with rehydration buffer were used for immobiline Drystrip (IPG pH3-10) passive rehydration step. The following running condition and voltage was used for 11 cm immobiline Drystrip gels on Ettan IPGphor 3 isoelectric focusing unit: Temperature 20 °C, Current 50 μA per strip, 300 V for 1 h, 3500 V for 1 h, 8000 V for 3 h, and 8000 V for 20,000 V-h (volt-hours). Firstly, Immobiline Drystrip gels (IPG) strip was incubated for 20 min in equilibration buffer (6 M urea, 0.375 M Tris HCl pH 8.8, 20% glycerol, 2%SDS,) with 150 mM DTT (Dithiothreitol) and secondly incubate was performed with gentle agitation of 135 mM Idoactamide in equilibration buffer for 10 min. Ultimately, IPG strip was detached and enclose it on to second dimension electrophoresis. The second dimension was achieved on 10% gel electrophoresis for 1 h and then gels (proteins) were stained with Coomassie Brilliant Blue as described previously^15^.

### Mass spectrometry (MS)

Spot proteins of interest were cut from gels and subjected to LC–Mass Spectrometry analysis by PhenoSwitch Bioscience Canada. Sample spots were dehydrated in 50 mM Tris + acetonitrile and then rehydrated with 10 mM Dithiothreitol at 65 °C for 15 min. Proteins were alkylated with 15 mM iodoacetamide at room temperature for 30 min. The gels were dehydrated to eliminate excess reagents and were rehydrated again in 50 mM Tris pH 8.0 plus 1 µg of Trypsin. The digestion was performed at 37 °C with shaking. The remaining peptides were purified by reversed phase solid phase extraction prior to LC-Mass Spectrometry analysis. Acquisition was carried out by ABSciex TripleTOF 5600 system. To control the instrument and data processing Analyst TF 1.7 software was used. Acquisition was performed in Information Source voltage (5.2 kV) was set and maintained at 225 °C. A reversed phase HALO C18-ES column, 0.3 μm intyrnal diameter and 2.7 μm particle size at 50 °C, was used for separation phase. Samples were added into a 5 μl loop followed the mobile phase (LC gradient phase) contains: 3% DMSO in water + 0.2% formic acid (solvent A) and 3% DMSO in EtOH + 0.2% v/v formic acid (solvent B) while flow rate was 10 μl/ min.

### Prediction of immune epitopes

The Immune Epitope Database and Analysis Resource (IEDB) and the BepiPred-2.0 server (IEDB, http://www.iedb.org) are used for predicting and analyzing epitopes from a protein sequence in the identified glycoproteins.

### Bioinformatics analysis

All proteins recognized by LC-Mass Spectrometry and ProteinPilot Software (https://www.sciex.com/) were analyzed for the presence of a potential N-linked and O-linked glycosylation sites. The sequences of each of the identified proteins were gained and Blast protein analysis (Basic Local Alignment Search Tool) was performed by NCBI server (https://www.ncbi.nlm.nih.gov/). The glycoproteins were identified using GlycoPP, a freely webserver available at http://www.imtech.res.in/raghava/glycopp/. Moreover, different Functional classes of potential glycoproteins were determined by Mycobrowser database (https://mycobrowser.epfl.ch/genes).

## Results

### Proteomics and ConA affinity chromatography

**Figure 1:**
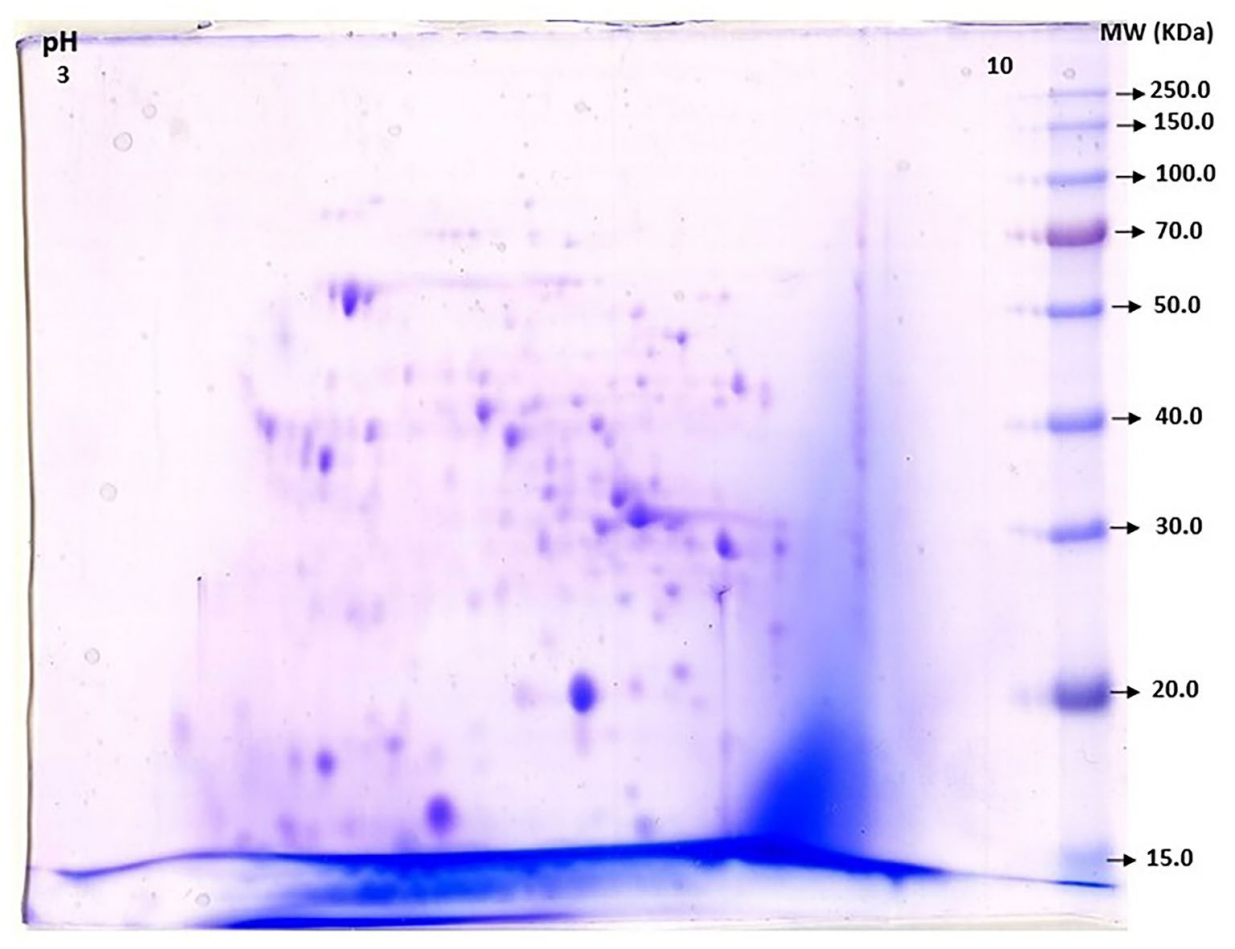
Glyco-protein spots present in two dimentional electrophoresis (2DE) patterns of *M. tuberculosis* strain (IPG strip pH3-10). The second dimension was achieved on 10% gel electrophoresis. Proteins were stained with Coomassie Brilliant Blue.

The purified contents obtained from ammonium sulfate precipitation of culture medium from *M. tuberculosis* were identified by their reactivity with ConA-Affinity Chromatography analysis. Purified glycoprotein bands (SDS-PAGE) in the Coomassie Brilliant Blue stained followed by ConA affinity chromatography are shown in Supplementary Fig. 1. The purified protein spots are released based on the increase in alpha methyl mannopyranoside concentration at multiple affinity peaks and the recognized glycoproteins evaluated by Two-dimensional gel electrophoresis (Fig. 1) and LC (liquid chromatography) Mass Spectrometry fractions. In some Con-A reactive spots or fraction cases, one-protein spots contain more than one peptide and because a ConA-Affinity Chromatography captured the protein contents, one would expect that most of the peptide spots correspond to glycoproteins, hence these cases to be confirm and identify again by mass spectrometry.

### Prediction of N- and O-glycosites in glycoprotein sequences

A total of 293 glycoproteins were identified using GlycoPP, a freely webserver available at http://www.imtech.res.in/raghava/glycopp/. The GlycoPP server allows for prediction of O- and N-glycosites in prokaryotic protein sequences in FASTA format. GlycoPP employs support vector machine established on position-specific scoring matrix, secondary structure, binary encoding, amino acid formation, and accessible surface area characters^16^. Predictions can be defined at threshold ranging from − 1.0 to 1.0 for optimizing scores. Threshold option is essential criteria for the stability of glycosites prediction procedures. In the present study, there were various thresholds and “0” selected as default threshold. Therefore, performance scales were estimated at several thresholds of scores ranging from − 1.0 to 1.0 and the perfect thresholds were selected for complementary optimization. Finally, the best performing pattern values were approved using an independent result of prokaryotic glycoproteins for decisive implementation at GlycoPP webserver^17^.

O-glycosylation constituted 6019 (90%) of the events identified and the remaining 697 sites (10%) were glycosylated at N residues events derived from 293 unique proteins in *M. tuberculosis* strains. Abundance of glycoproteins and glycosylation in *M. tuberculosis* membrane and secretory proteins and the number of N- and O-glycosylation events identified are displayed in Table 1. The positions of Asparagine and serin (or threonine) residues were considered as N and O glycosylation, respectively. For instance, the glycoprotein of Rv0440, which contains 540 amino acids, has 18 events, O-glycolysation equal to 3.33% and 4 events N glycolysation (0.7%) of the length of the proteins. Out of 293 unique identified proteins, at least 116 glycosylation events occurred with score greater than 1.

**Table 1:** Mycobacterial glycoproteins, type of glycosylation and their role in pathogenesis.

Gene	Proteins	Protein length (aa)^a^	O-glyc^b^ N (%)	N-glyc^c^ N (%)	Functional category
*Rv 0440*	Chaperonin 2 **groEL2**	540	18 (3.33)	4 (0.70)	Virulence, detoxification, adaptation
*Rv0685*	Elongation factor **TuOS**	396	10 (2.52)	4 (1.01)	Information pathways
*Rv0896*	Citrate synthase **gltA2**	431	22 (5.10)	4 (0.93)	Intermediary metabolism respiration
*Rv1479*	regulatory protein **MoxR1**	377	21 (5.60)	0 (0.00)	Regulatory proteins, transcriptional mechanism
*Rv3877*	ESX-1 secretion **espA**	392	33 (8.42)	4 (1.02)	Cell wall and cell processes, secretion system
*Rv0282*	ESX-3 secretion **eccA3**	631	31 (4.90)	1 (0.10)	Cell wall and cell processes, secretion system
*Rv3873*	PPE family	368	43 (11.7)	0 (0.00)	PE/PPE, immunomodulatory
*Rv2430c*	ESX-5 secretion- **EspG5**	194	10 (5.10)	2 (1.03)	PE/PPE, protein PPE41
*Rv3864*	ESX-1 secretion-** EspE**	402	24 (5.60)	3 (0.75)	Cell wall and cell processes
*Rv3881c*	ESX-1 secretion- **EspB**	460	23 (5.00)	2 (0.43)	Cell wall and cell processes
*Rv3532*	PPE family **PPE61**	406	43 (10.6)	3 (0.70)	PE/PPE
*Rv0475*	Heparin-binding **hbhA**	199	6, (3.02)	2 (1.00)	Cell wall and cell processes
*Rv2159c*	Conserved protein	344	20 (5.81)	1 (0.29)	Conserved hypotheticals
*Rv0173*	Mce lipoprotein **Lprk**	390	21 (5.38)	5 (1.28)	Cell wall and cell processes
*Rv0169*	Mce lipoprotein **Mce1A**	454	22 (4.84)	2 (0.44)	Cell wall and cell processes
*Rv3499c*	Mce lipoprotein **Mce4A**	400	29 (7.25)	4 (1.00)	Cell wall and cell processes
*Rv0170*	Mce lipoprotein **Mce1B**	346	13(3.76)	3(0.87)	Cell wall and cell processes
*Rv0174*	Mce lipoprotein **Mce1F**	515	32 (6.21)	7 (1.36)	Cell wall and cell processes
*Rv0593*	Mce lipoprotein **Mce2E**	402	26 (6.40)	5 (1.24)	Cell wall and cell processes
*Rv0583*	Lipoprotein **LpqN**	228	27 (11.8)	2 (0.11)	Cell wall and cell processes
*Rv0411c*	LipoproteinG **GlnH**	328	13 (3.96)	2 (0.61)	Cell wall and cell processes
*Rv1418*	Lipoprotein **LprH**	228	15 (96.58)	3 (1.32)	Cell wall and cell processes
*Rv1270c*	Lipoprotein **LprA**	244	25 (10.2)	4 (1.70)	Cell wall and cell processes

Descriptive of functional specification	Number of glycolytic proteins N (%)				
Conserved hypothetical	31(10.6)				
Intermediary metabolism and respiration	130 (44.3)				
Cell wall and cell processes	34 (11.6)				
PE/PPE proteins	3 (1.0)				
Lipid metabolism	41 (14.0)				
Regulatory systems	10 (3.40)				
Virulence, detoxyfication and adaptation	23 (7.90)				
Information pathways	19 (6.50)				
Unknown functions	2 (0.70)				
	Total: 293 (100)				

^a^Amino acids.

^b^Number of O-glycosylation.

^c^Number of N-glycosylation.

### Retrieving glycoprotein functions

As shown in Table 1, the identified spot proteins correspond to wide range of putative proteins stratified in different functional categories. The complete list of the *M. tuberculosis* membrane and secretory glycoproteins described in Supplementary material (Table S1). According to a functional specification of the reported mass spectrometry, GlycoPP webserver (http://www.imtech.res.in/raghava/glyopp) and Mycobrowser database (https://mycobrowser.epfl.ch/genes), the identified spots include 293 proteins from different functional classes: proteins of conserved hypothetical function 31(10.6%), proteins that are predicted to be involved in intermediary metabolism and respiration function 130 (44.3%), proteins involved in the cell wall and cell processes 34 (11.6%), PE/PPE proteins 3 (1.0%), proteins involved in lipid metabolism 41 (14.0%), proteins with a role in regulatory systems 10 (3.4%), proteins involved in virulence, detoxyfication and adaptation 23 (7.9%), proteins that are predicted to be involved in information pathways 19 (6.5%) and unknown functions 2 (0.7%). The MCE-family of proteins as a glycolipoproteins identified in the present study. A total of 169 glycosylation events (143 O-glyco and 26 N-glyc0) were identified on proteins expressed from the *M. tuberculosis* Mce-family lipoprotein.

### Immune epitope identification and immunogenicity

The immunogenicity of the identified glycoproteins was investigated using Mass spectrometry and the Immune Epitope Database and Analysis Resource (IEDB, http://www.iedb.org). There is a relatively high propensity of the glycosylation sites and location of continuous epitopes or antigenic determinant sites in the recognized glycoproteins with score greater than or equal to 6%. In other words, these glycolysis amino acids are placed in the position of immunogenic protein epitopes. List of the *M. tuberculosis* identified glycoproteins with score greater than 6% and predicted pepetides based on residue scores by IEDB analysis resource are depicted in Table 2. Probable conserved transmembrane protein (Rv0954), LpqN (Rv0583), PPE68 (Rv3873), Phosphate-binding protein (Rv0932c), PPE61 (Rv3532) and LprA (Rv1270c), had the highest glycosylation percentage value with 13.86%, 11.84%, 11.68%, 11.1%, 10.59% and10.2%, respectively (Table 2). Moreover**,** as shown in Table 2, Predicting B-cell epitope from protein sequences was made using the BepiPred-2.0, a web server for predicting B-cell epitopes from antigen sequences. BepiPred-2.0 is based on a random forest algorithm trained on epitopes and non-epitope amino acids determined from crystal structures (Jespersen 2017) and on a large collection of linear epitopes downloaded from the IEDB database. The Percentile compatibility prediction between glycosylation sites and antigenic determinat sites is performed afterwards.

**Table 2:** Glycoproteins with a glycosylation percentage greater than 6% and predicted peptides based on residue Scores by Immune database analysis resource (http://www.iedb.org).

Gene	Protein description	Glyc^a^ (%)	*O-glyc*^b^ (N)	Epitop^c^ (N)	Predicted peptides^d^		Percentile compatibility^e^ (%)
					Start (amino acid)	End (amino acid)	
Rv3873	PPE68	11.68	43	4	(54, 164, 263, 297)	(58, 249, 288, 365)	60.5
Rv0934	Phosphate-binding protein	9.0	34	15	(25, 87, 108, 138, 155, 189, 200, 255, 269, 285, 322, 344, 354, 359, 362)	(51, 89, 118, 147, 176, 189, 233, 261, 276, 307, 330, 352, 357, 360, 363)	53.0
Rv1078	Proline-rich antigen homolog	9.58	23	6	(1, 123, 130, 166, 187, 226)	(90, 123, 131, 174, 194, 227)	87.0
Rv2890	Ribosomal protein S2	6.97	20	5	(7, 18, 91, 106, 227)	(7, 27, 95, 156, 283)	60.0
Rv1679	Dehydrogenase, FadE16	9.38	35	18	(1, 16, 32, 49, 87, 104, 113, 126, 143, 159, 172, 191, 208, 243, 293, 312, 344, 346)	(6, 30, 32, 58, 97, 106, 121, 139, 145, 168, 181, 198, 216, 249, 302, 313, 344, 357)	54.3
Rv1860	Alanin and prolin rich (Apa)	6.46	21	12	(3, 34, 127, 133, 145, 175, 177, 198, 210, 227, 243, 2267)	(10, 118, 1321, 136, 162, 175, 191, 207, 223, 238, 257, 278, )	66.7
Rv0954	34 kDa antigenic protein	13.86	42	5	(1, 65, 100, 122, 160)	(35, 80, 100, 130, 300)	95.2
Rv0838	d-alanyl-dipeptidase	7.81	20	8	(25, 85, 141, 171, 193, 209, 228, 238)	(71, 97, 159, 187, 207, 210, 233, 250)	95.0
Rv1502	Uncharacterized protein	6.02	18	7	(13, 60, 108, 125, 168, 188, 242)	(20, 83, 113, 143, 175, 207, 263)	50.0
Rv2560	Uncharacterized protein	7.38	24	8	(1, 79, 84, 127, 181, 238, 252, 301)	(76, 81, 84, 148, 136, 249, 259, 301)	66.7
Rv3418c	10 kDa chaperonin, groS	6.0	6	5	(5, 18, 32, 49, 72)	(9, 24, 39, 60, 78)	50.0
Rv3532	PPE family protein PPE61	10.59	43	21	(10, 32, 38, 59, 62, 64, 72, 82, 96, 106, 144, 157, 166, 240, 266, 272, 286, 311, 315, 331, 356)	(26, 35, 46, 60, 62, 66, 72, 94, 98, 113, 153, 164, 201, 240, 266, 273, 299, 313, 329, 348, 389)	65.0
Rv2799	Probable membrane protein	8.13	17	7	(5, 40, 90, 111, 129, 152, 183)	(10, 84.109, 111, 145, 156, 205)	64.7
Rv0583c	Probable conserved lipoprotein, LpqN	11.84	27	7	(22, 69, 90, 112, 142, 175, 207)	(59, 81, 102, 116, 166, 181, 215)	77.8
Rv0932c	Phosphate-binding protein	11.1	41	14	(6, 25, 70, 78, 103, 141, 157, 190, 202, 255, 261, 281, 322, 346)	(6, 47, 70, 86, 122, 150, 177, 193, 233, 255, 268, 307, 329, 354)	60.97
Rv2890c	30S ribosomal protein	6.97	20	5	(7, 18, 91, 106, 227)	(7, 27, 95, 156, 283)	60.0
Rv1543	Uncharacterized oxidoreductase	6.16	21	13	(8, 15, 18, 40, 88, 95, 98, 118, 145, 199, 246, 274, 304)	(9, 16, 30, 51, 93, 95, 103, 118, 160, 201, 259, 279, 338)	61.9
Rv1270c	Lipoprotein LprA	10.2	25	13	(27, 55, 57, 72, 102, 120, 144, 146, 158, 179, 211, 222, 233)	(45, 55, 60, 92, 107, 139, 144, 148, 167, 198, 212, 222, 239, )	60.0
Rv1297	Transcription termination factor	6.31	38	20	(4, 42, 47, 50, 52, 54, 67, 259, 276, 297, 390, 392, 457, 479, 499, 524, 537, 567, 579, 592)	(40,44,48,50,52,66,223,265,287,330,390,393,467,485,503,528,550,569,584,599)	73.6

^a^Glycosylation percentage.

^b^Number of O-glycosylation.

^c^Number of epitope or antigenic determinant.

^d^Predicted peptides based on residue scores (Bepipred Linear Epitope Prediction 2.0 Results).

^e^Percentile compatibility between glycosylation sites and antigenic determinant sites.

## Discussion

Glycosylation is the most sufficient post-translational polypeptide modification in many cellular events such as cell–cell interactions, signaling, balancing the protein structure maturation, recognition and regulation of the catalytic enzymes activity^18–21^. Mainly, the study of the glycoproteome and characteristics of glycoproteins by different analytical techniques such as lectins (ConA affinity chromatography) and mass spectrometry (MS) can lead to the detect of biomarkers related to immunogenicity of pathogens, since determination of protein glycosylation can apply as means of diagnostic evaluation in infectious diseases^22^.

Glycoprotein Rv1886 (FbpB) has a mycolyltransferase activity^7^. This glycoprotein (Ag85) is a protein that may conserve survival of *M. tuberculosis* in intracellular parts of host cells and help to keep the *M. tuberculosis* cell wall stability by catalyzing the transfer of mycolic acids to cell wall arabinogalactan using the synthesis of the virulence factor TDM (trehalose6,6′-dimycolate)^7^. Moreover, the expression of Ag85 glycoprotein (FbpB and FbpC) can stimulate proliferation and differentiation of T cell and B cell antigens in tuberculosis patients and may have an application as a TB diagnostic test^7,23^. A crystal structure of RV0129c (fbpC), and RV1886 (FbpB), which are part of antigen 85 complex (Ag85) disclose probable mycolyltransferase active site and conserved fibronectin-binding sites, which are being explored as a possible drug target^24^.

Mycobacterial pathogens use the ESX-1 secretion system to escape the macrophage phagosome and transport protein substrates that mediate crucial interactions with the host. The ESX-1 specialized secretion system is essential for virulence and bacterial access to the host cytosol^25,26^. It may be that after they are secreted from pathogenic mycobacteria, ESX-1 and ESX-3 secretion system have opposing activities and induces phagosome membrane lysis^27^. Pathogenic *Mycobacteria* are responsible for the secretion of five different type VII secretion systems, which play obvious roles for bacterial survival and growth, including ESX-1 to ESX-5, share various attributes concerning genome structure, dimensions, antigenic characteristics, and vaccine capability but the molecules noticeably have very different function in bacterial physiology^24,28–30^. Although, the ESX-1 secretion system is present in most *Mycobacteria*, the ESX-5 system surprisingly, is limited to the subclass of slow-growing species which contains most pathogenic *Mycobacteria*. ESX-5 is also a crucial secretion system needed for nutrient uptake and cell membrane permeability. Furthermore, it secretes a huge number of substrates, including PE and PPE proteins, which play a role in evasion of host immune responses and immunomodulation^28^. This study determined a number of cell wall, membrane and associated membrane glycoproteins involved in cell process biosynthesis and drug efflux pumps including ESX 1–5 secretion system proteins, which are *M. tuberculosis* vaccine potential and drug targets. Glycoproteins associated with drug efflux pumps, include proteins like the eccA3 (Rv0282), espA (Rv3877), EsPE (Rv3864), EsPB (Rv3881c), EspG5 (Rv2430c), PPE68 (Rv3873) and PPE61 (Rv3532), (Tables 1 and 2).

It is relevant that the highly glycosylated *M. tuberculosis* proteins identified in the present work corresponded to Lipoproteins, a functionally several class of mycobacterial membrane proteins involved in cell invasion, colonization, evasion of host defense, cell biogenesis, adhesion, immunomodulation and transport over the membrane^7,9,31^. These include proteins like the lipoprotein LprA (Rv1270), LprH (Rv1418), GlnH (Rv0411c), LpqN (Rv05830, Mce lipoprotein Lprk (Rv0173), Mce1A (Rv0169), Mce2E (Rv0593), Mce4A (Rv3499c), Mce1B (Rv0170) and Mce1F (Rv0174) (Table 1).

Lipoprotein LprA *M. tuberculosis* is a glycosylated lipoprotein with strong TLR2 (Toll-like receptor 2) agonist functions. LprA induces cytokine responses, innate immunity and regulates APC activity of macrophages and dendritic cells. Its primary effect may potentially to help drive immune responses, but it may also induce homeostatic down-regulatory mechanisms like reduction of macrophage APC function that may enable evasion of immune surveillance by organisms and increased expression of CD40, CD80, and MHC-II^12,32,33^.

Rv1418 is a putative lipoprotein LprH Contains N-terminal signal sequence and properly positioned prokaryotic lipoprotein lipid attachment site. In a study by Skerry et al., to determine the role of probable TLR2 activating lipoproteins on mycobacterial-mediated HIV infectivity of CD4+ T cells, it was shown that the upregulation of *M. bovis* BCG lipoproteins, including LprF, LprH, LprI, LprP, LprQ, MPT83, and PhoS1, by *M. smegmatis* results greater ex vivo HIV infection of human PBMC CD4+ T cells. The potentiality of these lipoproteins to increase the HIV infectivity of CD4+ cells is inverted by chemical inhibitors of TLR2 signaling. This study revealed that lipoproteins expressed by virulent mycobacteria can activate immune pathways that increase T-cell sensitivity to HIV^34^.

Mce lipoproteins are conserved hypotheticals proteins encoded by mce operons and play a crucial role in the entry of Mycobacteria within the mammalian cell and their survival in epithelial cells and phagocytes. Mce-family proteins have the ability to preserve of cell surface properties in Mycobacteria pathogenesis by inhibiting alveolar macrophage activity and also can be associated with granuloma formation, invasion or long-term existence of Mtb bacilli in host cells^35,36^. Here, we identified the properties of the various *M. tuberculosis* Mce-family lipoprotein complexes, *mce1-4,* (Table 1) Which are involved in the lipid transport across the cell envelope such as import of mycolic or fatty acids and cholesterol. Mce1 has been shown to transport mycolic acids and fatty acids, while Mce4 just imports cholesterol^37^. It is reported that the Mce1 and Mce4 transporters are consist of subunits that provide substrate specificity and proteins that couple lipid transport to ATP hydrolysis. Therefore, potent chemical inhibitors of Mce-family lipoprotein would be predicted to block *M. tuberculosis* capability to use various key lipid nutrients concurrently while negatively impacting bacterial fitness, which may improve antibiotic treatment options for tuberculosis^38^.

This study identified a number of proteins with predicted N-terminal signal peptide specified that these are targeted to the secretory pathways as well as various glycoproteins belonging to the ESX secretion systems and to proline and glutamic acid (PE), and Pro–Pro–Glu (PPE, PE-PPE) families (Rv3873, Rv3532, Rv1078, Rv3877, Rv0282, Rv2430c, Rv3864, Rv3881c) (Supplementary Table 1). These glycoproteins represent various functions that heightened the virulence capability of *M. tuberculosis* intensely by modulating immune responses, whereby affecting immune system-mediated clearance of (mycobacterial) pathogen^39^.

Rv2430c (PPE41, EspG5) is a member of the *M. tuberculosis* PE/PPE families, which are important for virulence, growth and immunogenicity across their cell envelope by type VII secretion (ESX) systems^40,41^. A cytosolic chaperone, PPE41, EspG5, is needed for suitable folding and solidity of the PE–PPE proteins and eventually their proper secretion. EspG_5_ interacts only with PPE41 and support for preventing PE–PPE heterodimer aggregation on the PPE proteins^42^. Previous studies have described that Rv2430c (PPE41) a member of the PPE gene family to induce a strong B cell response, referring to the immunodominant nature of the protein^43–46^. Recent studies have displayed that the ESX system contributes to PE/PPE protein export. Furthermore, the ESX secretion-associated protein G (EspG), the homolog of the ESX system, distinguishes its related to PE/PPE protein, conserving it in a stable configuration and promoting^47^.

PPE68 (Rv3873) is a major antigenic PPE protein encoded by *M. tuberculosis* RD1 region as a vigorous stimulator of peripheral blood mononuclear cells collected from TB patients and BCG vaccinated healthy subjects. This PPE family protein is an immunogenic product and is localized in the membrane and the cell wall fractions of mycobacteria^48^. Based on current studies, PPE68 (Rv3873), as a potential virulence factor and significant immunogenic components of the PPE protein family is required for *M. tuberculosis* pathogenesis during infection^47,49^. The PE and PPE proteins are supposed to accomplish wide‐ranging roles in virulence and immune modulation. Apart from their distinguished role in preservation of granuloma, the modulation of TLR-mediated immune response by PE and PPE proteins have role in diverse cell processes. Thus, these proteins contain various immunogenic epitopes and are therefore of major interest for the development of novel tuberculosis vaccines^50,51^.

The present study aimed to analyze the immunogenicity of the identified glycoproteins in order to introduce likely antigenic protein candidates and epitopes to be used for the development of a new TB vaccine or therapeutic strategies against active tuberculosis. This research explores a relatively high propensity of the glycosylation sites and position of immunogenic epitopes or antigenic determinant sites in the recognized glycoproteins. Glycoproteins with a glycosylation percentage greater than 6% and predicted peptides based on residue Scores by Immune database analysis resource (http://www.iedb.org) and Bepipred Linear Epitope Prediction 2.0 are shown in Table 2. Moreover, percentile compatibility between glycosylation sites and antigenic determinant sites were also predicted as probable antigens in identified proteins. Percentile compatibility value varied from 53.0 (Rv0934) to 95.2 (Rv0954). These recognized glycoproteins with percentile compatibility greater than 50% had a higher antigenicity value and wide‐ranging roles in virulence and immune modulation. The glycosylation of proteins is known to adapt immunogenicity or pathogenicity and play a significant role in *M. tuberculosis* adaptive processes^7^. Therefore, the present study results that show high percentile compatibility between glycosylation sites and antigenic determinant to predict the immunogenicity of the identified peptides could be considered these glycoproteins that are relevant for diagnostics as well as for drug and vaccine discovery**.**

Taken together, our study discloses the existence of a number of glycoproteins that play *M. tuberculosis* survival, antigenicity and Immunogenicity. These contain proteins involved in host pathogen interaction, biosynthesis and transport of *M. tuberculosis* cell envelope, and drug efflux pumps, which are appealing for TB vaccine development and therapeutic strategies for treatment of tuberculosis.

### Supplementary Information

Supplementary Figure 1.

Supplementary Table S1.

## Data Availability

All data that support all the experimental findings in this article is available in the paper and supplementary Table.
