# Supplementary material for: A potent subset of Mycobacterium tuberculosis glycoproteins as relevant candidates for vaccine and therapeutic target
Source: Sci Rep. 2023 Dec 14;13:22194. doi: 10.1038/s41598-023-49665-2 (PMC10719292; doi:10.1038/s41598-023-49665-2)
Supplement: Supplementary file 1 — Supplementary Figure 1. [file 41598_2023_49665_MOESM1_ESM.docx]

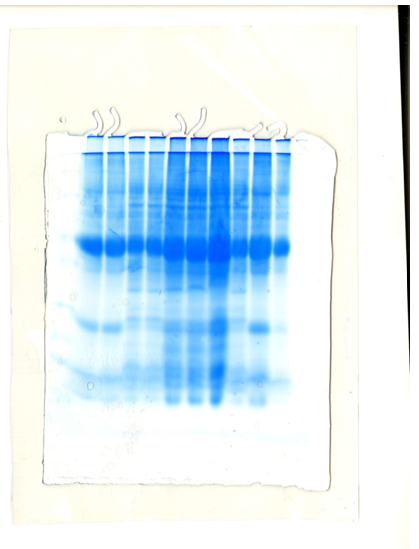


**10**

**9**

**8**

**7**

**6**

**5**

**4**

**3**

**2**

**1**

116.0

14.4

18.4

25.0

66.2

35.0

45.0

**MW(KDa)**

**Supplementary Figure 1:** Purified glycoprotein banding patterns (One dimensional gel electrophoresis) followed by ConA affinity chromatography on poly-acrylamide gel (10%) with Coomassie blue staining. Lanes 1-4 *Mycobacterium tuberculosis* glycoprotein patterns, Lanes 5-10 control samples (*Mycobacterium tuberculosis* strains H37Rv). Protein molecular weight markers are included: 116.0, 66.2, 45.0, 35.0, 25.0, 18.4, and 14.4 KDa.
