# Supplementary material for: A potent subset of Mycobacterium tuberculosis glycoproteins as relevant candidates for vaccine and therapeutic target
Source: Sci Rep. 2023 Dec 14;13:22194. doi: 10.1038/s41598-023-49665-2 (PMC10719292; doi:10.1038/s41598-023-49665-2)
Supplement: Supplementary file 2 — Supplementary Table S1. [file 41598_2023_49665_MOESM2_ESM.doc]

Supplementary Information

Table S1: *Mycobacterium tuberculosis* membrane and secretory glycoproteins were identified by Concanavalin Affinity

Chromatography and LC-Mass spectrometry

| Purified glycosylated proteins | Glycosylation number and percentage and their Functional category |
| --- | --- |
| Rv044060 kDa chaperonin 2 OS groEL2 Protein length == 540 amino acids | O-glycol(18 , 3.33%): 29, 49, 54, 164, 150, 155, 188, 199, 226, 258, 294, 300, 337, 356, 479, 499, 506, 528 N-glycol(4 ,0,74%): 36, 263, 468, 503virulence, detoxification, adaptation |
| Rv0242cProbable 3-oxoacyl-[acyl-carrier protein] reductaseFabGProtein length == 454 amino acids | O-glycol(27 , 5.95%): 6, 7, 11, 16, 20, 45, 82, 94, 157, 158, 164, 186, 195, 201, 219, 247, 257, 262, 335, 346, 347, 362, 363, 371, 395, 436, 440 N-glycol(4 , 0.88%):15, 294, 295, 333lipid metabolism |
| Rv1392S-adenosylmethionine synthase OSmetKProtein length == 403amino acids | O-glycol(17 , 4.21%):2, 14, 26, 40, 60, 62, 85, 105, 114, 129, 146, 271, 284, 289, 294, 340, 375 N-glycol(3 , 0.74%):98, 144, 246intermediary metabolism and respiration |
| Rv0685 Elongation factor Tu OStuf Protein length == 396 amino acids | O-glycol(10 , 2.52%): 17, 64, 96, 223, 230, 259, 301, 337, 385, 392 N-glycol(4 , 1.01%):44, 66, 239, 360informationpathways |
| Rv0694Putative L-lactate dehydrogenase OSlldDProtein length == 396 amino acids | O-glycol(16 , 4.04%): 32, 66, 73, 79, 108, 162, 190, 236, 271, 274,276, 286, 289, 297, 360, 374 N-glycol(1, 0.25%):277intermediary metabolism and respiration |
| Rv0350 Chaperone protein DnaK OS dnaK Protein length == 625 amino acids | O-glycol(28 , 4.48%): 17, 30, 33, 38, 78, 92, 194, 220, 246, 247, 250, 252, 259, 289, 295, 300, 305, 391, 401, 402, 409, 434, 479, 558, 574, 589, 592, 608 N-glycol(5 , 0.8%):29, 197, 336, 340, 389virulence, detoxification, adaptation |
| Rv1479 Probable transcriptional regulatory protein MoxR1Protein length == 377 amino acids | O-glycol(21 , 5.57%): 2, 3, 16, 21, 23, 42, 98, 100, 116, 128, 152, 164, 169, 174, 211, 234, 278, 285, 289, 363,375 N-glycol(0)regulatory proteins |
| Rv2416c N-acetyltransferase Eis Protein length == 402 amino acids | O-glycol(16 , 3.98%): 23, 25, 42, 56, 111, 121, 245, 287, 288, 318, 324, 367, 368, 380, 392, 398 N-glycol(0)virulence, detoxification, adaptation |
| Rv0896 Citrate synthase 1gltA2 Protein length == 431 amino acids | O-glycol(22 , 5.10%): 4, 40, 43, 44, 52, 57, 58, 60, 138, 145, 182, 237, 239, 247, 253, 254, 256, 284, 338, 384, 408, 418 N-glycol(4 , 0.93%): 142, 235, 250, 260intermediary metabolism and respiration |
| Rv1133c 5-methyltetrahydropteroyltriglutamate--homocysteine metE Protein length == 759 amino acids | O-glycol(40 , 5.27%): 2, 15, 39, 54, 56, 65, 72, 80, 92, 95, 231, 237, 261, 276, 317, 320, 324, 325, 326, 328, 380, 387, 409, 422, 435, 436, 439, 443, 444, 510, 517, 520, 568, 635, 636, 639, 669, 701, 706, 713, N-glycol(3, 0.4%):238, 690, 748intermediary metabolism and respiration |
| Rv0951 Succinate--CoA ligase [ADP-forming] subunit betasucCProtein length == 381amino acids | O-glycol(10 , 2.58%): 19, 27, 36, 118, 168, 172, 263, 321, 327, 386 N-glycol(2 , 0.52% ):142, 314intermediary metabolism and respiration |
| Rv1436Glyceraldehyde-3-phosphate dehydrogenase gapProtein length == 339 amino acids | O-glycol(8 , 2.36%): 54, 70, 103, 127, 154, 215, 244, 288 N-glycol(1 , 0.3% ):109intermediary metabolism and respiration |
| Rv3457cDNA-directed RNA polymerase subunit alpharpoAProtein length == 347 amino acids | O-glycol(14 , 4.03%): 44, 45, 52, 53, 86, 106, 155, 166, 203, 210, 246, 311, 314, 331 N-glycol(4 , 1.15%):17, 120 , 152, 200informationpathways |
| Rv3274c Probable acyl-CoA dehydrogenase fadE25Protein length == 389 amino acids | O-glycol(21 , 5.4%): 9, 24, 57, 58, 76, 112, 141, 167, 172, 191, 254, 283, 285, 286, 310, 326, 334, 341, 342, 369, 384 N-glycol(1 , 0.26%): lipid metabolism |
| Rv1074cProbable beta-ketoacyl CoA thiolase FadA3Protein length == 405amino acids | O-glycol(26 , 6.42%): 8, 9, 12, 21, 64, 84, 91, 92, 93, 97, 114, 119, 130, 146, 183, 226, 241, 254, 271, 274, 281, 289, 290 , 293, 352, 388 N-glycol(3 , 0.74% ):127, 250, 350lipid metabolism |
| Rv0155Probable NAD(P) transhydrogenase (Subunit alpha) PntAaProtein length == 366amino acids | O-glycol(27 , 7.38%):8, 17, 59, 64, 106, 137, 142,152, 154,155,162, 163, 167, 172, 196,211, 222, 250, 257, 258, 268, 282,291, 303, 310, 326, 366 N-glycol(2 , 0.55%): 140, 332intermediary metabolism and respiration |
| Rv1023Enolase,EnoProtein length == 429amino acids | O-glycol(22 , 5.13%):15, 39, 42, 43, 124, 157, 173, 198, 214, 215, 222, 252, 260, 262, 267, 300, 350, 365, 368, 382, 387, 393 N-glycol(3 , 0.7%):18, 108, 147intermediary metabolism and respiration |
| Rv1310ATP synthase subunit betaatpDProtein length == 486amino acids | O-glycol(29 , 5.97%):16, 17, 19, 20, 27, 93, 130, 195, 196, 224, 275, 278, 279, 286,306, 307, 308, 312, 314, 315, 341, 350, 363, 364, 370, 424, 434, 439, 481 N-glycol(1 , 0.2%):71intermediary metabolism and respiration |
| Rv3045NADP-dependent alcohol dehydrogenase CadhCProtein length =346amino acids | O-glycol(13 , 3.76%):2, 11, 19, 70, 93, 118, 126, 176, 207, 221, 287, 291, 299 N-glycol(1 , 0.29%):292intermediary metabolism and respiration |
| Rv2427cGamma-glutamyl phosphate reductaseproAProtein length =415 amino acids | O-glycol(16 , 3.86%):7, 34, 71, 139, 158, 159, 160, 164, 174, 190, 312, 343, 366, 367, 384, 401 N-glycol(7, 1.69%):82, 165, 237, 257, 267, 331, 364intermediary metabolism and respiration |
| Rv1843cUncharacterized oxidoreductaseguaB1Protein length =479 amino acids | O-glycol(27 , 5.64%)26, 38, 42, 44, 45, 54, 108, 198, 207, 212, 247, 255, 263, 278, 305, 309, 317, 354, 360, 368, 389, 397, 416, 418, 438, 442, 465 N-glycol(3 , 0.63%):24, 52, 355intermediary metabolism and respiration |
| Rv3417c60 kDa chaperonin 1, groEL1 , Protein length =539 amino acids | O-glycol(24 , 4.45%):49, 54, 75, 127, 133, 138, 141, 148, 151, 165,174, 179, 188, 199, 241, 294, 356, 426, 444, 470, 482, 502, 508, 516 N-glycol(4 , 0.74%):263,300, 467, 506virulence, detoxification, adaptation |
| Rv3028cElectron transfer flavoprotein subunit alphaetfAProtein length =318 amino acids | O-glycol(20 , 6.29%):19, 24, 42, 84, 85, 95, 111, 129, 145, 168, 199, 210, 215, 223, 237, 249, 254, 264, 275, 278, N-glycol(3 , 0.94%):142, 177, 213intermediary metabolism and respiration |
| Rv2951cPhthiodiolone/phenolphthiodiolonedimycocerosates ketoreductase Rv2951c Protein length =381 amino acids | O-glycol(14 , 3.67%):25, 31, 37, 51, 54, 55, 87, 106, 160, 204, 231, 244, 253, 295 N-glycol(4 , 1.05%):102, 161, 353, 358intermediary metabolism and respiration |
| Rv2215Dihydrolipoyllysine-residue acetyltransferase dlaTProtein length =553 amino acids | O-glycol(26 , 4.70%):4, 46, 50, 57, 66, 100, 113, 148, 165, 169, 179, 228, 245, 263, 265, 317, 318, 322, 328, 417, 450, 459, 464, 466, 470, 477 N-glycol(4 , 0.72%):330, 371, 393, 506intermediary metabolism and respiration |
| Rv1475cAconitate hydrataseA, canProtein length =943 amino acids | O-glycol(47 , 4.98%):8, 54, 57, 90, 175, 201, 205, 207, 210, 237, 249, 301, 306, 307, 327, 367, 385, 399, 402, 451, 478, 482, 483, 511, 512, 547, 552, 560, 571, 634, 641, 682, 683, 705, 716, 718, 723, 750, 780, 821, 824, 851, 868, 870, 871, 910, 942 N-glycol(15 , 1.59%):46, 50, 55, 111, 213, 299, 360, 481, 577, 591, 681, 703, 768, 889, 938intermediary metabolism and respiration |
| Rv3001cKetol-acid reductoisomerase (NADP(+))ilvCProtein length =333 amino acids | O-glycol(4 , 1.20%):24, 36, 251, 259 N-glycol(0):intermediary metabolism and respiration |
| Rv22453-oxoacyl-[acyl-carrier-protein] synthase 1kasAProtein length =416 amino acids | O-glycol(31 , 7.45%):2, 5, 6, 13, 17, 20, 23, 24, 26, 41, 70, 81, 97, 100, 114, 138, 166, 169, 172, 173, 175, 211, 217, 250, 271, 272,294, 301, 341, 346, 401 N-glycol(2 , 0.48%):8, 400350lipid metabolism |
| Rv3248cAdenosylhomocysteinase ahcY Protein length =495 amino acids | O-glycol(17 , 3.43%):12, 65, 74, 97, 216, 220, 221, 302, 336, 376, 395, 398, 403,414, 418, 422, 424 N-glycol(7 , 1.41%):17, 94, 241, 243, 253, 412, 427intermediary metabolism and respiration |
| Rv2890c30S ribosomal protein S2 rpsBProtein length =287 amino acids | O-glycol(20 , 6.97%):60, 72, 105, 159, 166, 209, 219, 256, 258, 260, 262, 264, 266, 268, 269, 270, 273, 283, 284, 287 N-glycol(1 , 0.35%):103informationpathways |
| Rv1543Uncharacterized oxidoreductase Rv1543Protein length =341amino acids | O-glycol(21 , 6.16%):18, 23, 26, 59, 62, 63, 106, 115, 193, 199, 210, 242, 246, 256, 267, 278, 279, 310, 311, 318, 329 N-glycol(3 , 0.88%):103, 166, 191lipid metabolism |
| Rv0173Possible Mce-family lipoprotein LprKProtein length =390amino acids | O-glycol(21 , 5.38%):29, 30, 37, 47, 53, 61, 68, 79, 107, 114, 146, 150, 151, 182, 215, 255, 262, 270, 334, 342, 347 N-glycol(5 , 1.28%):67, 87, 169, 253, 324cell wall and cell processes |
| Rv1488Uncharacterized protein Rv1488Protein length =381amino acids | O-glycol(13 , 3.41%):48, 73, 91, 108, 118, 137, 167, 173, 298, 325, 347, 353, 380 N-glycol(0):cell wall and cell processes |
| Rv0859Putative acyltransferase fadAProtein length =403amino acids | O-glycol(20 , 4.96%):60, 77, 82, 83, 94, 108, 121, 123, 129, 183, 190, 213, 257, 258, 271, 281, 289, 292, 387, 398 N-glycol(3 , 0.74%):20, 198, 256lipid metabolism |
| Rv14833-oxoacyl-[acyl-carrier-protein] synthase 2 ,kasBProtein length =247amino acids | O-glycol(16 , 6.48%):17, 21, 45, 49, 65, 124, 140, 142, 144, 156, 166, 172, 222, 226, 230, 233 N-glycol(3 , 1.21%): 24, 88, 175lipid metabolism |
| Rv0831cConserved proteinRv0831cProtein length =271amino acids | O-glycol(9 , 3.32%):57, 68, 97, 107, 122, 187, 210, 224, 229 N-glycol(3 , 1.11%):45, 151, 203conserved hypotheticals |
| Rv0655Probable ribonucleotide transport ATP-binding protein mklProtein length =359amino acids | O-glycol(16 , 4.46%):6, 9, 18, 19, 36, 39, 56, 62, 64, 72, 83, 165, 192, 199, 274, 309 N-glycol(1 , 0.27%):264cell wall and cell processes |
| Rv0934Phosphate-binding protein PstS,Protein length =374 amino acids | O-glycol(34, 9.09%):26, 30, 32, 35, 41, 44, 45, 48, 49, 52, 56, 59, 80, 82, 86, 88, 128, 186, 192, 210, 215, 216, 236, 241, 269, 280, 290, 292, 299, 328, 341, 347, 373, 374 N-glycol(3 , 0.8%):146, 229, 268cell wall and cell processes |
| Rv3679Putative ATPaseRv3679Protein length =340 amino acids | O-glycol(20 , 5.88%):4, 5, 6, 7, 10, 11, 16, 19, 27, 35, 36, 81, 126, 192, 204, 236, 264, 293, 296, 319 N-glycol(1 , 0.29%):202cell wall and cell processes |
| Rv1240Malate dehydrogenaseMdhProtein length =329 amino acids | O-glycol(43 , 13.07%):2, 4, 11, 20, 27, 29, 67, 73, 83, 100, 111, 130, 136, 143, 154, 157, 166, 172, 177, 183, 189, 191,223, 238, 239, 242, 245, 247, 258, 265, 270, 273, 282, 283, 287, 288, 294, 297, 305, 312, 313, 321, 324 N-glycol(3 , 0.912%):106, 187, 292intermediary metabolism and respiration |
| Rv2629Uncharacterized proteinRv2629Protein length =374 amino acids | O-glycol(17 , 4.55%):3, 24, 79, 127, 141, 156, 168, 169, 204, 210, 242, 254, 268, 302, 307, 371, 374 N-glycol(1 , 0.27%):86conserved hypotheticals |
| Rv2995c3-isopropylmalate dehydrogenaseleuBProtein length =336 amino acids | O-glycol(16 , 4.75%):34, 45, 52, 75, 107, 110, 124, 129, 148, 151, 214, 264, 272, 290, 325, 326 N-glycol(3 , 0.89%):112, 141, 262intermediary metabolism and respiration |
| Rv1437Phosphoglycerate kinasepgkProtein length =412 amino acids | O-glycol(25 , 6.07%):2, 15, 44, 111, 148, 154, 166, 195, 196, 225, 235, 243, 284, 288, 307, 320, 339, 350, 356, 363, 378, 381, 382, 395, 408 N-glycol(5 , 1.21%):26, 131, 317, 324, 371intermediary metabolism and respiration |
| Rv0046cInositol-3-phosphate synthaseino1Protein length =367 amino acids | O-glycol(10 , 2.77%): 6, 13, 28, 90, 99, 108, 200, 311, 314,341 N-glycol(3 , 0.81%):88, 89, 170intermediary metabolism and respiration |
| Rv25894-aminobutyrate aminotransferase gabTProtein length =449 amino acids | O-glycol(30 , 6.68%):3, 7, 12, 19, 23, 31, 32, 68, 69, 73, 74, 112, 120, 125, 139, 141, 164, 167,172, 266, 271, 292, 303, 306, 323, 390, 398, 403, 414, 429 N-glycol(5 , 1.11%):72, 124, 222, 313, 327intermediary metabolism and respiration |
| Rv3140Probable acyl-CoA dehydrogenase FadE23 , Protein length =401 amino acids | O-glycol(30 , 7.48%):57, 88, 113, 134, 142, 169, 183, 191, 217, 250, 275, 285, 294, 332, 343, 355, 356, 380, 396 N-glycol(0):lipid metabolism |
| Rv1771L-gulono-1,4-lactone dehydrogenase, Rv1771Protein length = 428 amino acids | O-glycol(25, 5.84%): 2, 6, 17, 24, 57, 112, 119, 121, 124, 132, 137, 142, 149, 162, 170, 234, 251, 256, 262, 276, 277, 278, 343, 350, 390 N-glycol(2 , 0.47%):7, 273intermediary metabolism and respiration |
| Rv0761cAlcohol dehydrogenase B , adhB, Protein length = 375 amino acids | O-glycol(19 , 5.1%):48, 69, 91, 119, 122, 136, 150, 172, 173, 177, 181, 208, 272, 277, 286, 290, 294, 313, 344N- glycol(4 , 1.06%):104, 309, 337, 360intermediary metabolism and respiration |
| Rv0524Glutamate-1-semialdehyde 2,1-aminomutase , hemL, Protein length = 462 amino acids | O-glycol(34 , 7.36%):3, 9, 27, 35, 41, 46, 51, 72, 98, 103, 135, 153, 169, 185, 188, 190, 191, 196, 197, 201, 208, 238,259, 273, 278, 280, 300, 331, 380, 393, 400, 404, 426, 434 N- glycol(1 , 0.21%):127intermediary metabolism and respiration |
| Rv0169Mce-family protein Mce1A,mce1A , Protein length = 454 amino acids | O-glycol(22 , 4.84%):17, 45, 48, 51, 78, 86, 113, 135, 158, 180, 189, 238, 251, 263, 313, 320, 325, 329, 334, 408, 421, 435 N- glycol(2 , 0.44%):128, 169, virulence, detoxification, adaptation |
| Rv2780Alanine dehydrogenase, ald, Protein length = 371 amino acids | O-glycol(20 , 5.39%):19, 43, 46, 60, 100, 110, 113, 114, 122, 134, 152, 178, 217, 253, 282, 309, 314, 344, 345, 357 N- glycol(6 , 1.61%):182, 188, 200, 252, 300, 315intermediary metabolism and respiration |
| Rv1612Tryptophan synthase beta chain, ,trpBProtein length = 410 amino acids | O-glycol(21, 5.12% ):2, 10, 15, 23, 51, 79, 84, 124, 133, 153, 177, 204, 213, 249, 278, 286, 289, 313, 315, 344, 390 N- glycol(4 , 0.97%):95, 103, 250, 388intermediary metabolism and respiration |
| Rv1079Cystathionine gamma-synthase, ,metB , Protein length = 388 amino acids | O-glycol(21 , 5.41%):6, 12, 29, 39, 40, 59, 63, 70, 84, 85, 90, 162, 177, 214, 266, 311, 328, 348, 354, 355, 358, N- glycol(1 , 0.25%):184intermediary metabolism and respiration |
| Rv1093Serine hydroxymethyltransferase 1, glyA1Protein length = 426 amino acids | O-glycol(21 , 4.93%): 2, 26, 94, 173, 213, 222, 234, 249, 288, 308, 311, 326, 359, 360, 366, 377, 388, 394, 395, 399, 422, N- glycol(2 , 0.47%):131, 139intermediary metabolism and respiration |
| Rv1308ATP synthase subunit alpha atpA , Protein length = 549 amino acids | O-glycol(27 , 4.92%):13, 20, 23, 26, 33, 49, 99, 126, 138, 179, 185, 195, 215, 233, 240, 242, 252, 254, 285, 316, 338, 343, 347, 375, 447, 514, 518 N- glycol(0):intermediary metabolism and respiration |
| Rv2397cPutative thiosulfate sulfur transferase, cysA1, Protein length = 351 amino acids | O-glycol(18 , 5.12%):39, 41, 58, 69, 125, 129, 136, 220, 233, 239, 244, 267, 270, 294,297, 302, 336, 350 N- glycol(2, 0.57%):64, 242cell wall and cell processes |
| Rv1323Probable acetyl-CoA acetyltransferase fadA4, Protein length = 389 amino acids | O-glycol(16 , 4.11%): 21, 23, 54, 86, 113, 126, 220, 239, 242, 244, 247, 283, 303, 323, 350, 367 N- glycol(2 , 0.514%):81, 123lipid metabolism |
| Rv2744c35 kDa protein , Rv2744c , Protein length = 270 amino acids | O-glycol(15 , 5.56%):45, 128, 156, 168, 170, 175, 183, 203, 209, 237, 249, 251, 256, 258, 259 N- glycol(1 , 0.37%):75conserved hypotheticals |
| Rv2202cAdenosine kinase ,adoK , Protein length = 324 amino acids | O-glycol(16 , 4.94%):2, 6, 11, 34, 105, 106, 115, 122, 134, 171, 178, 223, 248, 265, 268, 276 N- glycol(1 , 0.31%):187intermediary metabolism and respiration |
| Rv2970cCarboxylic ester hydrolase, LipN,Protein length = 376 amino acids | O-glycol(19 , 5.05%):49, 53, 72, 89, 113, 129, 200, 216, 222, 254, 257, 259, 295, 301, 303, 334, 343, 358, 367, N- glycol(2 , 0.53%):220, 350intermediary metabolism and respiration |
| Rv3410cUncharacterized oxidoreductaseguaB3Protein length = 375 amino acids | O-glycol(23, 6.13%):17, 20, 34, 35, 58, 104, 105, 136, 137, 142, 169, 175, 187, 210, 221, 227, 228, 235, 240, 272, 295, 313, 338 , N- glycol(2 , 0.53%): 75, 119 intermediary metabolism and respiration |
| Rv2623Universal stress proteinRv2623Protein length = 297 amino acids | O-glycol((17 , 5.72%): 2, 3, 6, 7, 17, 45, 90, 93, 103, 131, 133, 134, 202, 212, 263, 276, 287N- glycol(1 , 0.34%): 5 virulence, detoxification, adaptation |
| Rv0475Heparin-binding hemagglutinin, ,hbhA , Protein length = 199 amino acids | O-glycol(6 , 3.02%): 46, 92, 111, 119,142,197 N- glycol(2 , 1.0%):6, 29cell wall and cell processes |
| Rv3169Conserved protein Rv3169 , Protein length = 374 amino acids | O-glycol(13 , 3.47%):25, 49, 83, 170, 178, 255, 261, 268, 269, 297, 300, 364, 370 N- glycol(0):conserved hypotheticals |
| Rv1908cCatalase-peroxidase, katG , Protein length = 740 amino acids | O-glycol(21 , 2.84%):12, 17, 87, 180, 211, 271, 275, 302, 303, 306, 308,314, 315, 346, 363, 374, 457, 460, 465, 470, 474,475, 481, 482, 486,530, 539, 564, 575, 579, 620, 625, 647, 654, 700 N- glycol(5 , 0.67%):51, 133, 138, 562, 637virulence, detoxification, adaptation |
| Rv2986cDNA-binding protein HU , hup , Protein length = 214 amino acids | O-glycol(7 , 3.27%):41, 65, 75, 90, 109, 132, 208 N- glycol(0):informationpathways |
| Rv0884cPhosphoserine aminotransferase ,serC , Protein length 376 amino acids | O-glycol(22 , 5.85%):13, 47, 48, 63, 109, 114, 117, 129, 130, 134, 155, 156, 168, 178, 179, 214, 219, 252, 284, 290, 298, 303 N- glycol(4 , 1.06%):119, 152, 247, 251intermediary metabolism and respiration |
| Rv163030S ribosomal protein, rpsA , Protein length 481 amino acids | O-glycol(14 , 2.91%):25, 31, 37, 51, 54, 55, 87, 106, 160, 204, 231, 244, 253, 295 N- glycol(1 0.2%):204informationpathways |
| Rv0668DNA-directed RNA polymerase subunit beta, rpoC , Protein length 1316 amino acids | O-glycol(56 , 4.25%):24, 102, 112, 137, 260, 305, 308, 392, 425, 428, 471,503, 567, 570, 596, 603, 609, 613, 654, 727, 730,801, 805, 808, 816, 838, 839, 853, 863, 870, 881, 919, 924, 958, 971,972, 977, 982, 985,1002, 1008, 1014, 1065, 1115, 1148, 1166, 1170, 1172, 1214, 1219, 1222, 1225, 1262, 1287, 1294, 1301 N- glycol(7 , 0.53%):5, 35, 283, 349,384, 810, 1239informationpathways |
| Rv3570cFlavin-dependent monooxygenase, oxygenase subunit HsaA,Protein length 394 amino acids | O-glycol(23 , 5.8%):2, 3, 77, 81, 82, 87, 113, 118, 119, 142, 143, 209, 217, 225, 235, 239, 240, 242, 333, 336, 344, 350, 392, N- glycol(4 , 1.01%):136, 140, 300, 351intermediary metabolism and respiration |
| Rv1297Transcription termination factor, Rho , Protein length 602 amino acids | O-glycol(38 , 6.31%):12, 18, 36, 57, 58, 97, 98, 108, 115, 117, 126, 134, 136, 142, 147, 177,209, 242, 254, 270, 296, 333, 353, 360, 405, 412,453, 456, 461, 466, 489, 498, 500, 501, 567, 595, 598, 601, N- glycol(3 , 0.49%):298, 451, 584informationpathways |
| Rv2141cConserved protein, Rv2141c , Protein length 448 amino acids | O-glycol(14 , 3.13%):12, 30, 35, 36, 62, 79, 106, 188, 226, 254, 256, 279, 353, 389 N- glycol(1, 0.22%):302intermediary metabolism and respiration |
| Rv3520cPutative coenzyme F420-dependent oxidoreductaseRv3520c , Protein length 347amino acids | O-glycol(20 , 5.76%): 36, 49, 56, 57, 65, 66, 71, 76, 84, 89, 100, 135, 138, 139, 149, 153, 199, 328, , 333, 336 N- glycol(0):intermediary metabolism and respiration |
| Rv3734cProbable diacyglycerol O-acyltransferase, tgs2 , Protein length 454amino acids | O-glycol(15 , 3.30%):9, 16, 63, 92, 156, 180, 195, 229, 241, 243, 328, 330, 350, 367, 430 N- glycol(6 , 1.32%):228, 304, 312, 402, 410, 416lipid metabolism |
| Rv0154cProbable acyl-CoA dehydrogenase, FadE2, Protein length 403amino acids | O-glycol(15, 3.72%):2, 63, 66, 83, 98, 127, 138, 149, 165, 192, 200, 241, 357, 396, 403 N- glycol(1 , 0.25%):158lipid metabolism |
| Rv2005cUniversal stress proteinRv2005c , Protein length 295amino acids | O-glycol(16 , 5.42%):90, 93, 98, 103, 108, 118, 119, 131, 134, 169, 184, 210, 253, 255, 263, 278 N- glycol(3 , 1.01%):35, 109, 279virulence, detoxification, adaptation |
| Rv0889cPutative citrate synthase 2, citA , Protein length 373 amino acids | O-glycol(15 , 4.02%):63, 77, 121, 126, 147, 152, 172, 181, 184, 191, 200, 207, 325, 329, 359 N- glycol(1 , 0.26%):179intermediary metabolism and respiration |
| Rv3722cAspartate aminotransferase, Rv3722c , Protein length 435 amino acids | O-glycol(22 , 5.05%):2, 5, 7, 40, 100, 111, 119, 146, 148, 191, 196, 254, 255, 256, 259, 266, 271, 286, 399, 419, 429, 430 N- glycol(3 , 0.69%):97, 98, 405conserved hypotheticals |
| Rv1869cProbable reductase,Rv1869c , Protein length 411 amino acids | O-glycol(13 , 3.16%): 3, 4, 6, 26, 60, 85, 89, 112, 122, 141, 230, 342, 366, N- glycol(2 , 0.48%):375, 411intermediary metabolism and respiration |
| Rv3692 Probable methanol dehydrogenase transcriptional regulatory protein,Rv3692Protein length 358 amino acids | O-glycol(17 , 4.75%):4, 6, 13, 32, 36, 47,71, 101, 173, 190, 223, 241, 282, 290, 295, 300, 303 N- glycol(0):regulatory proteins |
| Rv2159cConserved protein Rv2159c , Protein length 344 amino acids | O-glycol(20 , 5.81%): 57, 78, 100, 108, 126, 128, 198, 217, 222, 228, 233, 241, 251, 260, 261, 306, 320, 327, 338, 343N- glycol(1 , 0.29%):341conserved hypotheticals |
| Rv3285Probable bifunctional protein acetyl-/propionyl-coenzymeaccA3 , Protein length 600 amino acids | O-glycol(24 , 4%): 3, 7, 66, 69, 83, 116, 119, 183, 199, 268, 371, 379, 383, 385, 393, 448, 456, 501, 530, 535, 572, 578, 580, 591N- glycol(4 ,0.67%):19, 86, 300, 502 lipid metabolism |
| Rv1309 ATP synthase gamma chain, atpG, Protein length 600 amino acids | O-glycol(21 , 6.88%):14, 30, 31, 42, 55, 83, 100, 135, 142, 155, 156, 175, 187, 191, 194, 196, 239, 249, 252, 288, 292 N- glycol(2, 0.65%):125, 267 intermediary metabolism and respiration |
| Rv1751 Probable oxidoreductase Rv1751 , Protein length 460 amino acids | O-glycol(17 , 3.7%): 12, 36, 42, 52, 53, 54, 119, 176, 189, 215, 248, 254, 310, 343, 374, 392, 430N- glycol(0):intermediary metabolism and respiration |
| Rv3329 Probable aminotransferase, Rv3329, Protein length 438 amino acids | O-glycol(17 , 3.9%):30, 60, 96, 126, 128, 134, 142, 155, 248, 269, 270, 273, 281, 300, 307, 384, 412 N- glycol(1 , 0.23%):314 intermediary metabolism and respiration |
| RV0352 Chaperone protein DnaJ 1, dnaJ1, Protein length 395 amino acids | O-glycol(22 , 5.6%):56, 90,120, 123, 124, 138, 173, 183, 190,200, 213, 217, 222, 224, 244, 274, 317, 330, 347, 350,382, 383 N- glycol(1, 0.25%):386 virulence, detoxification, adaptation |
| RV0530 Conserved protein , Rv0530 , Protein length 405 amino acids | O-glycol(26 , 6.41%):9, 16, 33, 47, 49, 68, 99, 110, 157, 166, 170, 196, 203,227, 228, 246, 279, 282, 288, 295, 296, 298, 304, 308, 403, 405 N- glycol(1 , 0.25%):126 conserved hypotheticals |
| Rv3340 Probable O-acetylhomoserinesulfhydrylase, MetC, Protein length 449 amino acids | O-glycol(24 , 5.34%):2, 5, 7, 43, 51, 74, 95, 96,117, 118, 167, 169,182, 250, 286, 301, 335, 349, 354, 392, 399, 409, 440,445 N- glycol(5, 1.11%):6, 72, 328, 369, 379 intermediary metabolism and respiration |
| Rv1464 Probable cysteine desulfurase, csd , Protein length 417 amino acids | O-glycol(24 , 5.75%):2, 7, 35, 39, 107, 113, 146, 156, 191, 195, 211, 230, 258, 265, 267, 272, 284, 287, 288,324, 335, 336, 342, 396 N- glycol(2 , 0.48%):6, 182 intermediary metabolism and respiration |
| Rv3048 Ribonucleoside-diphosphate reductase subunit beta, nrdF2Protein length 324 amino acids | O-glycol(16, 4.94% ):12, 54, 61, 66, 77, 82, 109, 111, 115, 159, 169, 186, 303, 305, 307, 308 N- glycol(2 , 0.62%): 15, 34 informationpathways |
| Rv2627c Uncharacterized protein , Rv2627c, Protein length 413 amino acids | O-glycol(18 , 4.36%):3, 4, 6, 18, 23, 90, 110, 141, 144, 152, 258, 271, 278, 330, 335, 380, 385, 412 N- glycol(2 , 0.48%):82, 268 conserved hypotheticals |
| Rv2373c Chaperone protein DnaJ 2 , dnaJ2, Protein length 382 amino acids | O-glycol(13 , 3.40%): 49, 105, 116, 118, 141, 157, 164, 185, 218, 303, 307, 310, 363N- glycol(1 , 0.26%):virulence, detoxification, adaptation |
| ESX-1 secretion-associated protein,espA, Protein length 392 amino acids | O-glycol(33 , 8.42%):2, 12, 130, 156, 183, 219, 223, 231, 240, 243, 244, 247, 250, 259, 261, 262, 269, 272,273275, 286, 293, 301, 325, 329, 333, 348, 349, 355, 356, 357, 361, 368 N- glycol(4 , 1.02%):69, 159, 228, 390 cell wall and cell processes |
| Rv1223 Probable serine (protease HtrA DEGP protein , Protein length 528 amino acids | O-glycol(31 , 5.87%): 63, 177, 210, 212, 217, 218, 239, 242, 255, 273, 280, 285, 326,346, 349, 354, 363,369, 387, 403, 407, 409, 411, 413, 445, 446, 448, 450, 455, 466, 527N- glycol(7 , 1.32%):268, 269, 278, 383, 386, 422, 451 intermediary metabolism and respiration |
| Rv3127 Uncharacterized protein Rv3127 , GN=Rv3127, Protein length 344 amino acids | O-glycol(15 , 4.36%):14, 18, 29, 32, 50, 57, 124, 183, 215, 251, 273, 276, 316, 338, 342 N- glycol(3 , 0.87%): 17, 39, 77 conserved hypotheticals |
| Rv1098c Fumarate hydratase class II , fumC, Protein length 474 amino acids | O-glycol(28 , 5.90%):7, 16, 42, 49, 66, 104, 107, 112, 118, 126, 142, 147, 152, 201, 229, 233, 260, 264, 278, 304, 318, 334, 380, 404, 407, 410, 414,471 N- glycol(5 , 1.05%):109, 140, 343, 359, 378 intermediary metabolism and respiration |
| Rv1480 Uncharacterized protein, Rv1480Protein length 317 amino acids | O-glycol(12 , 3.79%):107, 129, 133, 150, 156, 163, 167, 203, 250, 257, 280, 290 N- glycol(1 , 0.315%):132 conserved hypotheticals |
| Rv0642c Hydroxymycolate synthase MmaA4Protein length 301 amino acids | O-glycol(16 , 5.31%):17, 21, 45, 49, 65, 124, 140, 142, 144, 156, 166, 172, 222,226, 230, 233, N- glycol(1 , 0.33%):122 lipid metabolism |
| Rv0002 Beta sliding clamp OS=Mycobacterium tuberculosis, dnaN Protein length 402 amino acids | O-glycol(28, 6.96%):6, 21, 43, 55, 57, 64, 75, 78, 82, 110, 149, 160, 194, 197, 222, 227, 230, 243, 249, 274, 309, 345, 346, 353, 357, 358, 369, 388N- glycol(4 , 1%):52, 95, 336, 379 informationpathways |
| Rv3029c Electron transfer flavoprotein subunit beta , etfBProtein length 266 amino acids | O-glycol(13, 4.89%):64, 67, 74, 82, 104, 114, 127, 128, 155, 229, 234, 235, 247 N- glycol(3 , 1.12%):40, 125, 185 intermediary metabolism and respiration |
| Rv2996c D-3-phosphoglycerate dehydrogenase , sera, Protein length 528 amino acids | O-glycol(23 , 4.35%):15, 51, 53, 85, 97, 101, 128, 132, 133, 135, 137, 221, 283, 293, 298, 374, 382, 396, 403, 410, 427, 436, 528 N- glycol(3 , 0.57%):93, 98, 407 intermediary metabolism and respiration |
| Rv0400 Acyl-CoA dehydrogenase, FadE7, Protein length 395 amino acids | O-glycol(20 , 5.06%):2, 41, 82,98, 102, 109, 117, 120, 178, 202, 209, 215, 222, 251, 267, 280, 347, 355, 376, 389 N- glycol(0):lipid metabolism |
| Rv0391 O-succinylhomoserinesulfhydrylase , metZ, Protein length 406 amino acids | O-glycol(24 , 5.91%):: 18, 36, 43, 44, 50, 68, 76, 94, 96, 104, 120, 140, 166, 168, 173, 216, 250,255, 276, 279, 315, 319, 365, 406N-glycol(2 , 0.49%):72, 277 intermediary metabolism and respiration |
| Rv1099c Fructose-1,6-bisphosphatase class 2 ,glpX, Protein length 362 amino acids | O-glycol(23 , 6.35%):2,6, 8, 9, 10, 15, 19, 74, 76, 126, 130, 144, 165, 223, 229, 240, 318, 321, 326, 328, 347, 352, 355 N- glycol(4 , 1.11%):73, 344 intermediary metabolism and respiration |
| Rv0811c Conserved protein GN=Rv0811c, Protein length 368 amino acids | O-glycol(12 , 3.26%):59, 60, 64, 71, 76, 90, 119, 132,229, 288, 299, 328 N- glycol(0):conserved hypotheticals |
| Rv2200c Cytochrome c oxidase subunit 2 , ATP synthase subunit b-delta ctaC , Protein length 363 amino acids | O-glycol(18 , 4.96%):2, 13, 21, 43, 45, 48,50, 79, 93, 129, 229, 230, 239, 249, 304, 314, 346, 352 N- glycol(3 , 0.83%):68, 161, 274 intermediary metabolism and respiration |
| Rv1307 OS=Mycobacterium tuberculosis, atpFH , Protein length 446amino acids | O-glycol(12 , 2.69%):2, 36, 55, 144, 161, 181, 182, 200,208, 242, 336, 421 N- glycol(2 , 0,45%):134, 264 intermediary metabolism and respiration |
| Rv1837c Malate synthase G OS=Mycobacterium tuberculosis, glcB, Protein length 741amino acids | O-glycol(35 , 4.72%):101, 102, 103, 111, 112, 179, 184, 186, 194, 208, 221, 226,229, 251, 268, 334, 353, 379, 392, 426, 464, 471, 482, 485, 506, 536, 538, 539, 544, 546, 641,654, 655, 661, 732 N- glycol(10 , 1.35%):22, 55, 213, 234, 290, 295, 340, 382, 387, 425 intermediary metabolism and respiration |
| Rv1448c Transaldolase OS=Mycobacterium tuberculosis, tal, Protein length 373amino acids | O-glycol(15 , 4.02%):12, 17, 46, 57, 61, 90, 155, 195, 197, 201, 212, 252, 272, 320, 370 N- glycol(2 , 0.54%):165, 296 intermediary metabolism and respiration |
| Rv0462 Dihydrolipoyl dehydrogenase, lpdC, Protein length 464 amino acids | O-glycol(13 , 2.8%):28, 72, 142, 146, 152, 153, 160, 172, 231, 237, 392, 442, 447 N- glycol(1 , 0.22%):209 intermediary metabolism and respiration |
| Rv3206c Probable adenylyltransferase/sulfurtransferase ,MoeZ, Protein length 392amino acids | O-glycol(12 3.06%):2, 3, 68, 100, 104, 202, 215, 229, 287, 300, 329, 352 N- glycol(3 , 0.77%):42, 125, 332 intermediary metabolism and respiration |
| Rv3804 Diacylglycerol acyltransferase/mycolyltransferase Ag85A, fbpASecreted antigen 85-a FbpA(mycolyl transferase 85A) (fibronectin-binding protein A) (antigen 85 complex A , Protein length 338 amino acids | O-glycol(26 , 7.69%):12, 29, 40, 45, 58, 60, 71, 76, 107, 119, 123, 137, 146, 161, 163, 169, 173, 174, 177, 193, 199, 260, 302, 306, 328, 331, N- glycol(1 , 0.3%):246 lipid metabolism |
| Rv3423c Alanine racemase OS=Mycobacterium tuberculosis , alr , Protein length 408 amino acids | O-glycol(26 , 6.37%): 14, 15, 20, 21, 22, 26, 29, 75, 80, 93, 106, 134, 151, 160, 177, 206, 216, 235, 238, 261, 276, 286, 294, 305, 392, 393 N- glycol(1 , 0.25%):236 intermediary metabolism and respiration |
| Rv0270 Probable fatty-acid-CoA ligaseFadD2 (Fatty-acid-CoA synthetase) , Protein length 560 amino acids | O-glycol(24 , 4.29%):13, 18, 39, 195, 197, 209, 210, 211, 218, 222, 228, 230, 233, 241, 244, 253, 286, 310, 335, 343, 369, 388, 429, 482 N- glycol(5 , 0.89%):3, 188, 365, 386, 547 lipid metabolism |
| Rv0694 Putative mycofactocin system heme/flavin oxidoreductase MftD,Protein length 396 amino acids | O-glycol(16 , 4.04%):32, 66, 73, 79, 108, 162, 190, 236, 271, 274, 276, 286, 289,297, 360, 374 N- glycol(1 , 0.25%):277 intermediary metabolism and respiration |
| Rv2212 Uncharacterized protein Rv2212 , Rv2212, Protein length 378amino acids | O-glycol(17, 4.5%):4, 38, 84, 99, 140, 147, 181, 201, 202, 206, 218, 225, 263, 347, 371, 373, 377, N- glycol(1, 0.26%):308 intermediary metabolism and respiration |
| Rv2981c D-alanine--D-alanine ligase, Ddl , Protein length 373 amino acids | O-glycol(20 , 5.36%):21, 27, 30, 33, 55, 78, 146, 177, 200, 201, 205, 208, 209, 254, 269, 338, 339, 341, 350, 364 , N- glycol(2, 0.54%):22, 37 cell wall and cell processes |
| Rv2298 Uncharacterized oxidoreductase, Rv2298, Protein length 323 amino acids | O-glycol(16 , 4.95%):13, 19, 24, 64, 83, 138, 150, 167, 198, 261, 272, 273, 289, 307, 308, 321, N- glycol(2, 0.62%):148, 168 conserved hypotheticals |
| Rv3255c Mannose-6-phosphate isomerase, manA , Protein length 408amino acids | O-glycol(17 , 4.17%):17, 23, 50, 71, 73, 154, 164, 214, 217, 225, 249, 278, 327, 350, 364,366, 373 N- glycol(2 , 0.49%):125, 246intermediary metabolism and respiration |
| Rv3389c 3-hydroxyacyl-thioester dehydratase Y , htdY, Protein length 290 amino acids | O-glycol(14 , 4.83%):24, 35, 41, 45, 82, 98,101, 137, 138, 192, 234, 235, 251, 257N- glycol(2, 69%):44, 232intermediary metabolism and respiration |
| Rv0243 Probable acetyl-CoA acyltransferase FadA2 (3-ketoacyl-CoA thiolase) , Protein length 440 amino acids | O-glycol(18 , 4.1%):9, 46, 128, 129,179, 182, 190, 263, 268, 271, 275, 289, 318, 384, 385, 395, 403, 425, N- glycol(6 , 1.36%):7, 211, 240, 266, 308, 382lipid metabolism |
| Rv0824c Putative acyl-[acyl-carrier-protein] desaturase, DesA1 , Protein length 338 amino acids | O-glycol(8 , 2.37%):81, 104, 142, 159, 194, 228, 322, 336N- glycol(2 , 0.59%):138, 217 lipid metabolism |
| Rv0952 Succinate--CoA ligase [ADP-forming] subunit alpha,sucD, Protein length 303 amino acids | O-glycol(25 , 8.25%):9, 20, 25, 28, 36, 49, 52, 76, 81, 117, 131, 143, 147, 152, 157, 185, 186, 198, 256, 265, 267, 268, 270, 292, 302 N- glycol(3 , 0.99%):124, 137, 155 intermediary metabolism and respiration |
| Rv1656 Ornithine carbamoyltransferase , argF, Protein length 307 amino acids | O-glycol(14 , 4.56%):32, 56, 73, 75, 76, 87, 152, 164, 171, 177, 201, 257, 283,307N- glycol(1 , 0.33%):121 intermediary metabolism and respiration |
| Rv3168 Putative aminoglycoside phosphotransferase , GN=Rv3168 , Protein length 378 amino acids | O-glycol(15 , 3.97%):17, 28, 32, 33, 44, 47, 51, 55, 62, 122, 195, 197, 319, 333, 344 N- glycol(1 , 0.26%):3 virulence, detoxification, adaptation, |
| Rv2244 Meromycolate extension acyl carrier protein , acpM, Protein length 115 amino acids | O-glycol(4 , 3.48%):4, 20, 25, 114 N- glycol(0):lipid metabolism |
| Rv0562 Probable polyphenol-diphosphate synthase GrcC1 , Protein length 335 amino acids | O-glycol(16 , 4.78%): 35, 45, 47, 67, 80, 111, 150, 171, 185, 198, 209, 230, 249, 259, 290, 332 N- glycol(2 , 0.6%):53, 168 intermediary metabolism and respiration |
| Rv0860 3-hydroxyacyl-CoA dehydrogenase,fadB , Protein length 720amino acids | O-glycol(36 , 5%):24, 37, 51, 59, 72, 135, 143, 156, 234, 235, 247, 278, 284, 323, 344, 368, 381, 382, 396, 440, 441, 446, 465, 506, 512, 534, 540, 613, 627, 628, 659, 666, 676, 679, 687, 692N- glycol(5 , 0.69%):31, 248, 439, 504, 663 lipid metabolism |
| Rv1923 Probable lipase, LipD, Protein length 446 amino acids | O-glycol(27 , 6.05%):13, 26, 29, 44, 68, 93, 96, 102, 140, 145, 193, 206, 236, 243, 266, 268, 302, 309, 327, 337, 357, 358, 375, 383, 385, 404, 414 N- glycol(1 , 0.22%):413 intermediary metabolism and respiration |
| Rv1131 2-methylcitrate synthase , prpC, Protein length 393 amino acids | O-glycol(20 , 5.1%):10, 39, 102, 191, 195, 200, 212, 213, 220, 221, 222, 224, 231, 289, 323, 359, 370, 379, 392 N- glycol(1, 0.22%):210 intermediary metabolism and respiration |
| Rv3846 Superoxide dismutase [Fe] , sodB, Protein length 207 amino acids | O-glycol(8 , 3.86%):57, 84, 105, 118, 119, 123, 132, 201 N- glycol(4 , 1.93%):62, 75, 135, 183 virulence, detoxification, adaptation |
| Rv3774 Possible enoyl-CoA hydratase EchA21(Enoylhydrase) , Protein length 274 amino acids | O-glycol(13 , 4.74%):20, 57, 59, 64, 113, 130, 138, 177, 209, 234, 253, 261, 272 N- glycol(1, 0.36%):62 lipid metabolism |
| Rv2889c Elongation factor Ts OS=Mycobacterium tuberculosis, tsf, Protein length 271 amino acids | O-glycol(4, 1.47%):111, 125, 152, 189, N- glycol(1 , 0.37%):245 informationpathways |
| Rv0800 Probable M18 family aminopeptidase 2 ,apeB , Protein length 433 amino acids | O-glycol(26 , 6.0%):4, 17, 57, 66, 69, 82, 131, 162, 210, 216, 220, 249, 252, 268, 270, 276, 280, 281, 304, 308, 379, 380, 389, 393, 419, 427 N- glycol(1 , 0.23%):218 intermediary metabolism and respiration |
| Rv3596c ATP-dependent Clp protease ATP-binding subunit ClpC1, Protein length 848 amino acids | O-glycol(30 , 3.54%):48, 61, 75, 155, 162, 164, 166, 168, 179, 223, 306, 324, 373, 437, 456, 492, 529, 546, 548, 555, 592, 620, 648, 666, 671, 674, 682, 736, 744, 839, N- glycol(8, 0.94%): 26, 101, 177, 210, 391, 490, 568, 806 intermediary metabolism and respiration |
| Rv2032 Putative NAD(P)H nitroreductaseacg ,acg, Protein length 331amino acids | O-glycol(19 , 5.74%):4, 7, 13, 23, 27, 55, 79, 80, 97, 145, 177, 187, 193, 199, 200, 203, 276, 281, 294 N- glycol(1 , 0.3%):26 conserved hypotheticals |
| Rv1071c Possible enoyl-CoA hydratase EchA9 (Enoylhydrase) , Protein length 345amino acids | O-glycol(22, 6.38%):2, 5, 10, 57, 121, 126, 132, 133, 168, 173,201, 206, 218, 235, 260, 262, 267, 294, 296, 299, 320, 328 N- glycol(0):lipid metabolism |
| Rv0906 Uncharacterized protein Rv0906 , GN=Rv0906, Protein length 372amino acids | O-glycol(15 , 4.03%):12, 14, 28, 36, 43, 82, 84, 113, 131, 232, 245, 261, 268, 309, 311 N- glycol(1 , 0.27%):239conserved hypotheticals |
| Rv3720 Probable fatty acid methyltransferaseRv3720, Protein length 420amino acids | O-glycol(18 , 4.28%):9, 11, 19, 24, 61, 91, 101, 157, 222, 255, 261, 262, 266, 303,305, 328, 329,374 N- glycol(2, 0.48%):293, 301 lipid metabolism |
| Rv2460c ATP-dependent Clp protease proteolytic subunit 2 , clpP2 , Protein length 214amino acids | O-glycol(16, 7.5%): 3, 6, 23, 65, 78, 84, 85, 92, 102, 110, 120, 138, 172, 202, 210, 213 N- glycol(1 , 0.47%):2 intermediary metabolism and respiration |
| Rv0707 30S ribosomal protein S3 ,rpsC, Protein length 274amino acids | O-glycol(17 , 6..09%): 15, 16, 69, 110, 124, 139, 154, 165, 231, 238,241, 243, 244, 246, 248, 271, 274 N- glycol(1 , 0.36%):107informationpathways |
| Rv1600 Histidinol-phosphate aminotransferase ,hisC , Protein length 380amino acids | O-glycol(20 , 5.26%):87, 102, 119, 126, 128, 134, 183, 208, 209, 212, 227, 264, 267, 278, 281, 297, 309, 349, 362, 375 N- glycol(3 , 0.78%): 100, 103, 176 intermediary metabolism and respiration |
| Rv2953 Trans-acting enoyl reductase , GN=Rv2953 , Protein length 418amino acids | O-glycol(30 , 7.17%):19, 23, 30, 32, 33, 42, 43, 132, 144, 177, 182, 190, 195, 197, 198, 245, 276, 284, 295, 321, 326, 344, 345, 346, 395, 365, 366, 373, 414, 415, N- glycol(1 , 0.24%):56 ):lipid metabolism |
| Rv0500 Pyrroline-5-carboxylate reductase, proC , Protein length 295amino acids | O-glycol(18 , 6.1%):15, 52, 57, 95, 108, 120, 138, 147, 155, 176, 179, 181, 218, 254, 255, 259, 260, 287 N- glycol(2 , 0.67%):64, 86 intermediary metabolism and respiration |
| Rv3651 Uncharacterized protein , GN=Rv3651 , Protein length 345amino acids | O-glycol(16 , 4.64%):32, 37, 50, 53, 61, 87, 118, 120, 122, 150, 177, 187, 254, 261, 287, 317 N- glycol(4 , 1.16%):125, 146, 148, 285 conserved hypotheticals |
| Rv0363c Fructose-bisphosphate aldolase , FbaProtein length = 344amino acids | O-glycol(10 , 2.9%):30, 46, 53, 61, 118, 133, 190, 255, 279, 325 N- glycol(3, 0.87% 19, 27, 210 intermediary metabolism and respiration |
| Rv3148 NADH-quinone oxidoreductase subunit D , nuoD, Protein length = 440amino acids | O-glycol(16 , 3.64%):2, 7, 32, 50, 89, 93, 143, 185, 233, 250, 340, 350, 379, 390, 405, 430 N- glycol(4 , 0.90%):106, 139, 184, 200 intermediary metabolism and respiration |
| Rv2222c Glutamine synthetase , glnA2 , Protein length = 446amino acids | O-glycol(27 , 6.05%):33, 55, 63, 67, 74, 83, 84, 85, 90, 98, 103, 105, 126, 182, 242, 268, 273, 286, 307, 310,317, 331, 339, 342, 397, 405, 433 N- glycol(3 , 0.67%):153, 345, 423 intermediary metabolism and respiration |
| Rv0066c Isocitrate dehydrogenase [NADP], icd2, Protein length = 515amino acids | O-glycol(28 , 5.44%):32, 49, 91, 101, 129, 130, 131, 173, 194, 205, 243, 246, 291, 293, 300, 331, 338, 352, 381, 384, 405, 423, 452, 466, 504, 507, 510, 513 N- glycol(4 , 0.78%):87, 220 241, 310 intermediary metabolism and respiration |
| Rv1001 Arginine deiminase, arcA , Protein length = 402amino acids | O-glycol(14 , 3.48%):9, 30, 46, 80, 111, 122, 134, 164, 165, 176, 234, 235, 381, 394 N- glycol(0):intermediary metabolism and respiration |
| Rv3499c Mce-family protein Mce4A tuberculosis, mce4A , Protein length = 400amino acids | O-glycol(29 , 7.25%):2, 6, 10, 25, 34, 39, 42, 44, 46, 47, 71, 74, 76, 88, 96, 106, 112, 122, 127, 135, 165, 168, 280, 315, 316, 317, 324, 337, 384 N- glycol(4 , 1.0%):97, 161, 242, 341 virulence, detoxification, adaptation |
| Rv3917c Probable chromosome-partitioning protein, ParB,, Protein length = 344amino acids | O-glycol(15 , 4.36%):21, 28, 32, 39, 41, 53, 66, 90, 104, 108, 110, 133, 217, 247, 305 N- glycol(1 , 0.29%):196cell wall and cell processes |
| Rv1324 Uncharacterized protein ,Rv1324, Protein length = 304amino acids | O-glycol(16 , 5.26%):19, 32, 33, 40, 43, 97, 131, 132, 148, 151, 162, 163, 166, 227, 231, 267 N- glycol(2 , 0.66%):28, 199 intermediary metabolism and respiration |
| Rv0809 Probable phosphoribosylformylglycinamidine CYCLO-ligase ,  purM , Protein length = 364amino acids | O-glycol(13 , 3.75%):14, 18, 73, 74, 152, 167, 197, 201, 261, 287, 293, 325, 333 N- glycol(1 , 0.27%):202 intermediary metabolism and respiration |
| Rv2540c Chorismate synthase, aroC , Protein length = 401amino acids | O-glycol(21 , 5.23%):6, 30, 61, 67, 80, 87, 137, 149, 165, 169, 173, 218, 238, 239, 246, 291, 303, 318, 374, 388, 400 N- glycol(0):intermediary metabolism and respiration |
| Rv2220 Glutamine synthetase , glnA1, Protein length = 478 amino acids | O-glycol(16 , 3.35%):40, 57, 121, 122, 127, 141, 143, 146, 159,209, 222, 259, 273, 280, 301, 343, 348, 356, 369, 372, 373 N- glycol(3 , 0.63%):149, 229, 346 intermediary metabolism and respiration |
| Rv2715 Uncharacterized protein Rv2715 s , GN=Rv2715Protein length = 341 amino acids | O-glycol(17 , 4.98%):36, 52, 106, 111, 135, 156, 184, 185, 207, 213, 265, 273, 295, 322, 325, 339, 341 N- glycol(2 , 0.59%):331, 336 intermediary metabolism and respiration |
| Rv3778c Uncharacterized protein Rv3778c , GN=Rv3778cProtein length = 398 amino acids | O-glycol(28 , 7.03%): 14, 33, 37, 39, 40, 45, 48, 49, 55, 59, 99, 100, 152, 157, 165, 167, 234, 243, 244, 249, 270, 280, 292, 295, 310, 331, 375, 382 N- glycol(3 , 0.75%):216, 237, 368 intermediary metabolism and respiration |
| Rv3684 Probable lyase , GN=Rv3684Protein length = 346 amino acids | O-glycol(29 , 8.38%):9, 12, 67, 72, 73, 75, 76, 79, 99, 100, 102, 109, 121, 182, 185, 186, 210, 217, 228, 259, 267, 277, 280, 298, 308, 315, 323, 342, 346N- glycol(2 , 0.58%): 65, 119 intermediary metabolism and respiration |
| Rv2868c 4-hydroxy-3-methylbut-2-en-1-yl diphosphate synthase (flavodoxin) , ispG , Protein length = 387 amino acids | O-glycol(16 , 4.13%):2, 14, 21, 33, 44, 52, 150, 170, 189, 227, 230, 245, 279, 282, 332, 377, 379, 381, 385, 387 N- glycol(3 , 0.78%):51, 318, 334 conserved hypotheticals |
| Rv2524c Probable fatty acid synthase Fas (Fatty acid synthetase),  Protein length = 3069 amino acids | O-glycol(162 , 5.3%):10, 17, 20, 43, 54, 115, 140, 189, 199, 228, 239, 274, 354, 370, 383, 391, 392, 418, 428, 429, 503, 513, 535, 543, 559, 593, 607, 616, 637, 645, 646, 647, 648, 650, 658, 666, 676, 677, 679, 691, 723, 754, 757, 759, 762, 881, 921, 976, 977, 1010, 1024, 1025,1039, 1066, 1069, 1079, 1101, 1106, 1140, 1142, 1149, 1158, 1162, 1182, 1184, 1198, 1204, 1227, 1247, 1256, 1264, 1266, 1280, 1317, 1322, 1354, 1386, 1390, 1407, 1413, 1449, 1490, 1509, 1510, 1524, 1531, 1549, 1567, 1659, 1666, 1745, 1752, 1762,1763, 1786, 1809,1848, 1871, 1908, 1939, 1947, 1952, 1955, 1960, 1967, 2006, 2042, 2081, 2087, 2097, 2122, 2125, 2132, 2148, 2200, 2205, 2230, 2255, 2291, 2298,2304, 2311, 2345, 2368, 2397, 2417, 2455, 2478, 2553, 2576, 2598, 2607, 2612, 2617, 2623, 2635, 2641, 2642, 2656, 2661, 2664, 2671, 2674, 2705, 2709, 2764, 2767, 2768, 2778, 2781, 2799, 2831, 2850, 2875, 2897, 2906, 2009, 2940, 2975, 2976, 3023 N- glycol(12, 0.39%):596, 752, 804, 1009, 1048, 1145, 1200, 1383, 1823, 2631, 2660, 2882 lipid metabolism |
| Rv1295 Threonine synthase OS=Mycobacterium tuberculosis, thrC , Protein length = 360 amino acids | O-glycol (19 , 5.28%):8, 48, 51, 92, 93, 96, 97, 99, 158, 160, 209, 226, 248, 254, 260, 268, 299, 302, 344 N- glycol(4 , 1.11%):63, 95, 166, 197 intermediary metabolism and respiration |
| Rv2299c Chaperone protein , HtpG, Protein length = 647 amino acids | O-glycol (20 , 3.1%):61, 150, 153, 157, 158, 175, 222, 225, 238, 246, 299, 375, 417, 418, 421, 424, 429, 449,511, 550 N- glycol(4 , 0.62%):28, 39, 350, 385virulence, detoxification, adaptation |
| Rv3464 dTDP-glucose 4,6-dehydratase ,rmlB, Protein length = 331amino acids | O-glycol(14, 4.22%): 6, 14, 19, 36, 65, 81, 119, 139, 144, 145, 148, 168, 197, 239 N- glycol(3 , 0.9%):142, 173, 311 intermediary metabolism and respiration |
| Rv1238 Trehalose import ATP-binding protein , SugC, Protein length = 393 amino acids | O-glycol(16, 4.07%):26, 39, 45, 57, 133, 136, 187, 237, 248, 253, 324, 344, 359, 383 N- glycol(4 , 1.02%): 10, 24, 69, 386 cell wall and cell processes |
| Rv1194 Conserved protein , GN=Rv1194c, Protein length = 421 amino acids | O-glycol( (20 , 4.75%):7, 106, 149, 157, 184, 201, 207, 230, 235, 242, 274, 297, 318, 329, 330, 339, 410, 411, 416, 418 N- glycol(2, 0.48%):23, 264 conserved hypotheticals |
| Rv3010c ATP-dependent 6-phosphofructokinase , pfkA , Protein length = 343 amino acids | O-glycol(15, 4.37%): 7, 25, 32, 33, 71, 108, 111, 114, 142, 148, 156, 185, 215, 248, 281N- glycol(0):intermediary metabolism and respiration |
| Rv2564 Uncharacterized ABC transporter ATP-binding protein , GN=Rv2564, Protein length = 330 amino acids | O-glycol(17 , 5.15%): 7, 15, 27, 34, 42, 61, 63, 98, 116, 145, 241, 251, 271, 290, 297, 300, 305 N- glycol(0):cell wall and cell processes |
| Rv2538c 3-dehydroquinate synthase , aroB, Protein length = 362 amino acids | O-glycol(4 , 1.1%):51, 125, 296, 306 N- glycol(2, 0.55%):148, 154 intermediary metabolism and respiration |
| Rv2258 Possible transcriptional regulatory protein , Rv2258c , Protein length = 353 amino acids | O-glycol(16 , 4.53%):2, 8, 21, 29, 39, 48, 70, 82, 95, 133, 182, 199, 204, 212, 306, 325N- glycol(2 , 0.57%):222, 346 regulatory proteins |
| Rv1094 Putative acyl-[acyl-carrier-protein] desaturase, DesA2, Protein length=275amino acids | O-glycol (4 , 1.45%): 59, 147, 268, 274 N-glycol(1, 0.36%):21 lipid metabolism |
| Rv0282 ESX-3 secretion system protein EccA3, eccA3 , Protein length=631amino acids | O-glycol (31 , 4.91%): 9, 23, 27, 66, 92, 102, 126, 150, 151, 165, 192, 218, 268, 289, 291, 297, 298, 299, 307, 359, 389, 439, 533, 538, 554, 558, 593, 598, 610, 617, 628 N-glycol(1 , 0.16%):281 cell wall and cell processes |
| Rv0468 3-hydroxybutyryl-CoA dehydrogenase , fadB2 , Protein length=286amino acids | O-glycol(16 , 5.59%): 2, 18, 24, 57, 62, 100, 121, 122, 123, 133, 170, 175, 189, 203, 279, 285 N-glycol(3 , 1.04%):120, 146, 194 lipid metabolism |
| Rv2226 Uncharacterized protein Rv2226 , GN=Rv2226 , Protein length=513amino acids | O-glycol(23 , 4.48%): 23, 28, 95, 97, 125, 126, 130, 153, 219, 220, 240, 267, 273, 278, 287, 349, 355, 377, 381, 402, 439, 455, 471 N-glycol(0): conserved hypotheticals |
| Rv0407 F420-dependent glucose-6-phosphate dehydrogenase , fgd1, Protein length=336amino acids | O-glycol(12, 3.57%): 34, 38, 54, 59, 73, 78, 92, 107,116, 195, 196, 261N-glycol(1 , 0.29%):82 intermediary metabolism and respiration |
| Rv0020c FHA domain-containing protein FhaA , fhaA, Protein length=527amino acids | O-glycol(24 , 4.55%): 3, 30, 50, 82, 112, 143, 160, 161, 206, 282, 301, 307, 332, 336, 363, 382, 408, 422, 431, 434, 437, 454, 470, 497 N-glycol(6 , 1.14%): 60, 144, 159, 492, 495, 501 regulatory proteins |
| Rv3801c Long-chain-fatty-acid--AMP ligase , FadD32,Protein length=637amino acids | O-glycol(41, 6.44%4, 27, 82, 137, 142, 145, 157, 194, 195, 197, 202, 232, 259, 281, 284, 286, 325, 328, 344, 349, 360, 361, 398, 415, 445, 455, 458, 474, 514, 527, 541, 597, 604, 605, 606, 622, 625, 626, 631, 635, 636 N-glycol(4 , 0.63%): 13, 210, 319, 380 lipid metabolism |
| Rv3130c Probable diacyl glycerol O-acyltransferase, tgs1, Protein length=463amino acids | O-glycol(26 , 5.62%): 53, 127, 153, 158, 160, 167, 168, 171, 173, 175, 190, 192, 193, 207, 215, 219, 261, 279, 287, 290, 295, 340, 373, 396, 453, 460 N-glycol(2, 0.43%): 184, 222 lipid metabolism |
| Rv0384c Chaperone protein , ClpB, Protein length=848amino acids | O-glycol(35 , 4.13%): 3, 7, 15, 22, 23, 75, 78, 84, 92, 93, 107, 117, 120, 132, 146, 151, 165, 244, 269, 282, 289, 343, 369, 383, 408, 543, 546, 583, 600, 609, 674, 720, 724, 839, 844 N-glycol(5, 0.6%): 5, 200, 201, 257, 837 virulence, detoxification, adaptation |
| Rv1267c Transcriptional regulatory protein , EmbR , Protein length=388amino acids | O-glycol(21, 5.41%): 4, 26, 30, 66, 74, 77, 104, 109, 135, 139, 188, 218, 265, 268, 269, 275, 281, 297, 359, 381, 384, N-glycol(3 , 0.77%):45, 47, 348 regulatory proteins |
| Rv1703c Probable catechol-O-methyl transferase , GN=Rv1703cProtein length=196amino acids | O-glycol(1, 0.5%): 143 N-glycol(2, 1.2%): 70, 73 intermediary metabolism and respiration |
| Rv3224 Possible iron-regulated short-chain dehydrogenase/reductase , GN=Rv3224 , Protein length=282amino acids | O-glycol(13, 4.6%): 7, 11, 14, 38, 51, 78, 82, 83, 98, 128, 194, 198, 249 N-glycol(4, 1.42%): 31, 96, 229, 275 intermediary metabolism and respiration |
| Rv2740 Epoxide hydrolase EphG , Protein length=149amino acids | O-glycol(4, 2.68%): 8, 14, 15, 60 N-glycol(0): virulence, detoxification, adaptation |
| Rv1106c 3 beta-hydroxysteroid dehydrogenase/Delta 5-->4-isomerase , GN=Rv1106c, , Protein length=370amino acids | O-glycol(15 , 4.05%): 11, 20, 43, 49, 79, 129, 133, 180, 186, 223, 244, 281, 321, 364, 367 N-glycol(5, 1.35%): 107, 132, 173, 227, 318, intermediary metabolism and respiration |
| Rv2294 Putative cystathionine beta-lyase , GN=Rv2294, Protein length=407amino acids | O-glycol(25 , 6.14%): 16, 19, 20, 117, 128, 155, 156, 160, 161, 163, 164, 180, 183, 203, 213, 234, 236, 271, 279, 284, 350,368, 369, 397, 402 N-glycol(4, 0.98%):173, 176, 240, 299intermediary metabolism and respiration |
| Rv2067c Uncharacterized protein Rv2067c , GN=Rv2067c, Protein length=407amino acids | O-glycol(16 , 3.93%):12, 31, 43, 75, 128, 195, 217, 242, 282, 313, 331, 345, 371, 375, 392, 406, N-glycol(1, 0.25%):291 conserved hypotheticals |
| Rv2841c Transcription termination/antitermination protein, NusA, protein Length = 347 amino acids | O-glycol(16 , 4.6%): 28, 70, 75, 85, 86, 123, 139, 142, 156, 218, 273, 279, 286, 340 N-glycol(0): information pathways |
| Rv0131c Probable acyl-CoA dehydrogenase , FadE1 , protein Length = 447 amino acids | O-glycol(17 , 3. 8%):56, 106, 115, 144, 155, 165, 183, 209, 217, 232, 237, 266, 279, 317, 390, 418, 422 N-glycol(1 , 0.22%):95lipid metabolism |
| Rv3562 Probable acyl-CoA dehydrogenase , FadE31, protein Length = 377 amino acids | O-glycol(24 , 5.37%):26, 29, 31, 54, 89, 93, 94, 105, 118, 128, 132, 144, 159, 160, 185, 208, 239, 240, 241, 247, 292, 297, 309, 342 N-glycol(4 , 1.06%):23, 91, 153, 242 lipid metabolism |
| Rv0379 Calcium dodecin , secE2 , , protein Length = 71 amino acids | O-glycol(3, 4.22%):12, 16, 49 N-glycol(0): cell wall and cell processes |
| Rv0972c Acyl-CoA dehydrogenase, fadE12 , protein Length = 388 amino acids | O-glycol(20, 5.15%):5, 21, 73, 84, 92, 122, 128, 141, 148, 160, 174, 251,274, 275, 332, 341, 359, 369, 381, 387 N-glycol(6 , 1.55%):25, 57, 96, 135, 242, 345 lipid metabolism |
| Rv2941 Long-chain-fatty-acid--AMP ligase , FadD28, protein Length = 580 amino acids | O-glycol(43, 7.41%):2, 42, 60, 61, 68, 106, 111, 112, 120, 121, 164, 171, 172, 174, 184, 203, 211, 246, 260, 266, 300, 307, 328, 340, 341, 349, 356, 358, 372, 431, 436, 444, 447, 493, 499, 515, 532, 535, 537, 541, 556, 557, 558 N-glycol(1, 0.17%): 471 lipid metabolism |
| Rv3227 3-phosphoshikimate 1-carboxyvinyltransferase OS, aroA , protein Length = 450 amino acids | O-glycol(37 , 8.22%):3, 8, 11, 24, 44, 45, 47, 52, 55, 79, 97, 114, 154, 165, 167, 168,172, 177, 192, 193, 196, 203, 217, 248, 253, 266, 289, 324, 327, 329, 332, 349, 369, 371, 436, 444, 448 N-glycol(1, 0.22%): 152 intermediary metabolism and respiration |
| Rv2207 Nicotinate-nucleotide—dimethylbenzimidazolephosphoribosyltransferase , cobT, protein Length = 361 amino acids | O-glycol(21, 5.81%):8, 9, 24, 34, 39, 44, 71, 74, 81, 136, 150, 177, 265, 288, 289, 333, 344, 345, 347, 354, 360 N-glycol(2 , 0.55%): 87, 176 intermediary metabolism and respiration |
| Rv2334 O-acetylserinesulfhydrylase cysK1protein Length = 310amino acids | O-glycol(14 , 4.51%): 8, 23, 72, 75, 116, 123, 166, 179, 184, 196, 211, 242, 267, 271 N-glycol(1 , 0.32%): 38 intermediary metabolism and respiration |
| Rv2031c Alpha-crystalline , hspX , protein Length = 144 amino acids | O-glycol(7, 4.86%): 4, 13, 19, 27, 37, 129, 142 N-glycol(0): virulence, detoxification, adaptation |
| Rv0341 Isoniazid-induced protein IniB , protein Length = 479 amino acids | O-glycol(34 , 7.1%): 3, 14, 20, 30, 31, 43, 62, 93, 96, 97, 105, 122, 123, 141, 160, 189, 232, 237, 249, 252, 255, 256, 262, 265, 273, 287, 316, 345, 385, 393, 404, 420, 429, 470 N-glycol(3 , 0.63%):205, 247, 263cell wall and cell processes |
| Rv1860 Alanine and proline-rich secreted protein , Apa, protein Length = 325 amino acids | O-glycol(21 , 6.46%): 25, 30, 49, 50, 53, 56, 57, 66, 131, 146, 147, 177, 183, 211, 218, 232, 240, 245, 282, 315, 316 N-glycol(2 , 0.62%): 161, 268 cell wall and cell processes |
| Rv1078 Proline-rich antigen homolog,pra , protein Length = 240 amino acids | O-glycol(23 , 9.58%): 10, 21, 35, 44, 45, 47, 67, 87, 89, 122, 123, 126, 127, 139, 142, 170, 173, 174, 178, 186, 188, 189, 234 N-glycol(1 , 0.42%): 163 conserved hypotheticals |
| Rv3873PPE family immunomodulatory, PPE68, protein Length = 368amino acids | O-glycol (43 , 11.68%): 33, 35, 51, 57, 61, 69, 79, 80, 84, 90, 97, 102, 115, 120, 122, 129, 172, 179, 182, 183, 191, 194, 195. 196, 206, 216, 230, 231, 234, 239, 257, 258, 261, 269, 272, 282, 293, 297, 316, 317, 319, 336, 339 N-glycol(0) PE/PPE |
| Rv0357c Adenylosuccinatesynthetase , purA, protein Length = 432 amino acids | O-glycol(16 , 3.70%): 47, 58, 66, 94, 128, 129, 239, 244, 252, 257, 270, 275, 300, 301, 382, 410 N-glycol(2 , 0.46%): 37, 242 intermediary metabolism and respiration |
| Rv2916c Signal recognition particle protein , ffh , protein Length = 525 amino acids | O-glycol(18 , 3.43%): 65, 66, 98, 101, 114, 130, 166, 169, 237, 238, 272, 410, 415, 432, 433, 452, 459, 463 N-glycol(3 , 0.57%):79, 399, 408 cell wall and cell processes |
| Rv1449c Transketolase , tkt , protein Length = 700 amino acids | O-glycol(40 , 5.71%): 8, 24, 51, 71, 82, 108, 116, 131, 132, 141, 149, 186, 190, 215, 223, 257, 261, 279, 381, 386, 399, 408, 409, 415, 422, 427, 434, 449, 468, 493, 510, 529, 543, 574, 604, 617, 639, 641, 666, 673 N-glycol(5 , 0.71%):43, 406, 527, 541, 700 intermediary metabolism and respiration |
| Rv2178 Phospho-2-dehydro-3-deoxyheptonate aldolase, AroG , protein Length = 462 amino acids | O-glycol(14 , 3.03%): 39, 62, 95, 136, 146, 187, 188, 189, 208, 336, 375, 376, 413, 438 N-glycol(4 , 0.86%): 2, 175, 327, 416 intermediary metabolism and respiration |
| Rv3330 D-alanyl-D-alanine carboxypeptidase, DacB1, protein Length = 405 amino acids | O-glycol(21 , 5.19% , 0.49%): 6, 8, 19, 42, 50, 67, 81, 85, 93, 104, 121, 210, 214, 216, 224, 226, 283, 286, 336, 340, 370 N-glycol(2): 133, 138 cell wall and cell processes |
| Rv3042c Phosphoserine phosphatase , SerB2, protein Length = 409 amino acids | O-glycol(14 , 3.42%): 12, 20, 21, 94, 106, 111, 136, 188, 213, 258, 261, 273, 327, 387 N-glycol(2 , 0.48%): 123, 295 intermediary metabolism and respiration |
| Rv0054 Single-stranded DNA-binding protein , ssb , protein Length = 164 amino acids | O-glycol(15 , 9.15%): 5, 24, 35, 80, 114, 121, 123, 128, 137, 138, 139, 141, 148, 152, 154 N-glycol(2 , 1.22%): 30, 118information pathways |
| Rv0014c Serine/threonine-protein kinase, PknB, protein Length = 626 amino acids | O-glycol(45 , 7.19%): 3, 5, 23, 77, 106, 111, 147, 166, 169, 171, 179, 184, 192, 197, 220, 223, 237, 244, 265, 298, 305, 325, 328, 357, 369, 392, 393, 408, 411, 421, 445, 457, 459, 460, 478, 481, 490, 521, 524, 527, 543, 549, 567, 612, 621 N-glycol(5 , 0.79%):67, 127, 258, 303, 419 regulatory proteins |
| Rv1679 dehydrogenase, FadE16 , protein Length = 373 amino acids | O-glycol(35 , 9.38%): 3, 12, 23, 54, 60, 67, 72, 73, 90, 104, 114, 118, 120, 127, 128, 130, 147, 157, 160, 177, 187, 196, 208, 237, 242, 245, 263, 279, 280, 294, 301, 323, 350, 355, 368 N-glycol(1 , 0.27%): 194 lipid metabolism |
| Rv0975c Probable acyl-CoA dehydrogenase , FadE13, protein Length = 382 amino acids | O-glycol(24 , 6.28%): 5, 88, 102, 127, 141, 156, 159, 160, 169, 173, 190, 209, 230, 233, 241, 252, 253, 277, 323, 348, 366, 371, 377, 382 N-glycol(0):lipid metabolism |
| Rv2241 Pyruvate dehydrogenase E1 component , aceE, protein Length = 901 amino acids | O-glycol(32 , 3.55%): 3, 22, 61, 107, 108, 111, 112, 128, 177, 194, 220, 239, 407, 492, 503, 535, 551, 564, 579, 582, 605, 612, 615, 632, 636, 639, 677, 738, 810, 841, 894, 895 N-glycol(7 , 0.78%):313, 351, 405, 575, 594, 686, 801 intermediary metabolism and respiration |
| Rv0490 Sensor-like histidine kinase , SenX3, protein Length = 410 amino acids | O-glycol(23 , 5.6%):5, 14, 30, 41, 49, 68, 117, 119, 122, 125, 171, 236, 239, 282, 288, 292, 295, 297, 336, 342, 368, 370, 409, N-glycol(2 , 0.49%): 164, 283 regulatory proteins |
| Rv1844c 6-phosphogluconate dehydrogenase, decarboxylating gnd1, protein Length = 485 amino acids | O-glycol(36 , 7.42%): 2, 3, 4, 6, 16, 23, 35, 42, 55, 62, 138, 149, 158, 161, 170, 180, 187, 279, 300, 302, 307, 310, 314, 328, 354, 391, 409, 413, 419, 420, 428, 433, 451, 462, 477, 481 N-glycol(3 , 0.62%): 40, 146, 196 intermediary metabolism and respiration |
| Rv1201c 2,3,4,5-tetrahydropyridine-2,6-dicarboxylate N-succinyltransferase , dapD, protein Length = 317 amino acids | O-glycol(32 , 10.1%): 2, 5, 32, 34, 36, 37, 39, 40, 45, 70, 74, 112, 117, 178, 196, 203, 212, 219, 225, 229, 233, 244, 249, 263, 266, 269, 273, 293, 295 N-glycol(4 , 1.26%): 103, 243, 292, 317 intermediary metabolism and respiration |
| Rv0932c Phosphate-binding protein, PstS 2, protein Length = 370 amino acids | O-glycol(41 , 11.11%): 6, 21, 27, 29, 30, 31, 32, 37, 38, 40, 51, 52, 54, 55, 84, 89, 101, 108, 115, 121, 143, 161, 183, 188, 193, 214, 216, 226, 232, 237, 238, 248, 263, 272, 273, 279, 296, 303, 329, 351, 370 N-glycol(8 , 2.16%): 28, 82, 94, 106, 157, 218, 229, 367 cell wall and cell processes |
| Rv0411c lipoprotein GlnH (GLNBP) , GlnH, protein Length = 328 amino acids | O-glycol(13 , 3.96%): 29, 44, 61, 112, 135, 143, 154, 176, 192, 211, 212, 213, 228 N-glycol(2 , 0.61%): 174, 292 cell wall and cell processes |
| Rv0721 30S ribosomal protein S5 , rpsE, protein Length = 220 amino acids | O-glycol(8 , 3.64%): 11, 28, 40, 53, 65, 131, 207, 213 N-glycol(0): information pathways |
| Rv2536 Probable conserved transmembrane protein , GN=Rv2536, protein Length = 230 amino acids | O-glycol(9 , 3.91%): 2, 33, 87, 103, 160, 164, 173, 185, 216 N-glycol(1 , 0.43%): 27 cell wall and cell processes |
| Rv1248c Multifunctional 2-oxoglutarate metabolism enzyme ,kgd, protein Length = 1231 amino acids | O-glycol(58 , 4.71%): 5, 6, 28, 46, 53, 57, 78, 93, 119, 126, 129, 158, 160, 189, 192, 196, 212, 223, 231, 246, 254, 256, 262, 268, 312, 324, 364, 509, 557, 570, 575, 585, 633, 640, 695, 770, 824, 827, 903, 913, 938, 941, 943, 952, 990, 997, 1026, 1040, 1042, 1048, 1050, 1119, 1120, 1203, 1208, 1211, 1212, 1214 N-glycol(10 , 0.81%): 3, 117, 361, 568, 604, 682, 714, 752, 856, 970 intermediary metabolism and respiration |
| Rv0170 Mce-family protein, Mce1B, protein Length = 346 amino acids | O-glycol(13 , 3.76%): 14, 40, 47, 58, 92, 94, 175, 188, 236, 252, 269, 332, 337 N-glycol(3 , 0.87%):234, 260, 303 virulence, detoxification, adaptation |
| Rv2130 L-cysteine:1D-myo-inositol 2-amino-2-deoxy-alpha-D-glucopyranoside ligase,mshC, protein Length = 414 amino acids | O-glycol(16, 3.86%): 24, 46, 176, 217, 220, 239, 243, 262, 270, 293, 295, 303, 313, 331, 347, 349N-glycol(1 , 0.24%): 81 intermediary metabolism and respiration |
| Rv3680 Probable anion transporter ATPase GN=Rv3680, protein Length = 386 amino acids | O-glycol(16 , 4.15%): 7, 34, 35, 49, 113, 125, 126, 177, 206, 223, 253, 274, 308, 315, 355, 374 N-glycol(2 , 0.52%): 283, 345 cell wall and cell processes |
| Rv2857c Probable short-chain type dehydrogenase/reductaseRv2857c, protein Length = 258 amino acids | O-glycol(13 , 5.04%): 16, 20, 36, 137, 141, 150, 153,158, 160, 168, 240, 251, 252 N-glycol(1 , 0.39%): 115 intermediary metabolism and respiration |
| Rv2430c ESX-5 secretion-associated protein EspG5 , PPE family protein PPE41, protein Length = 194 amino acids | O-glycol(10 , 5.15%): 12, 23, 48, 129, 176, 184, 186, 187, 189, 194 N-glycol(2, 1.03%): 11, 46 PE/PPE(PPE41) |
| Rv0954 34 kDa antigenic protein homolog , GN=Rv0954, protein Length = 303 amino acids | O-glycol(42 , 13.86%): 4, 18, 23, 34, 55, 63, 74, 77, 101, 119, 126, 129, 130, 156, 160, 204, 210, 218, 224, 225, 226, 228, 230, 232,235, 246, 248, 251, 256, 257, 260, 264, 266, 272, 275, 277, 281, 287, 290, 295, 296, 297 N-glycol(3 , 0.99%): 123, 285, 288cell wall and cell processes |
| Rv1063c Uncharacterized NTE family protein, Rv1063c, protein Length = 360 amino acids | O-glycol(24 , 6.67%): 12, 13, 14, 23, 51, 52, 88, 90, 121, 132, 147, 190, 207, 217, 219, 225, 235, 250, 252,254, 276, 291, 297, 322 N-glycol(3 , 0.83%): 192, 205, 289 conserved hypotheticals |
| Rv1886 acyltransferase/mycolyltransferase Ag85B , fbpB, protein Length = 325 amino acids | O-glycol(27, 8.31%): 37, 42, 55, 57, 68, 73, 104, 107, 116, 117, 120, 134, 143, 150, 158, 160, 166, 170, 171, 188, 190, 196, 202, 223, 303, 320, 321 N-glycol(6 , 1.85%): 71, 89, 243, 253, 291, 299 lipid metabolism |
| Rv0174 Mce-family protein , Mce1F , protein Length = 515 amino acids | O-glycol(32 , 6.21%): 17, 33, 49, 64, 69, 86, 102, 114, 122, 123, 128, 134, 179, 207, 211, 231, 238, 246, 255, 260, 298, 301, 303, 333, 337, 344, 425, 454, 472, 473, 476, 480, 483, 500, 501, 502, 515 N-glycol(7 , 1.36%): 87, 142, 190, 200, 235, 427, 498 virulence, detoxification, adaptation |
| Rv3133c DNA-binding transcriptional activator DevR/DosR, protein Length = 217 amino acids | O-glycol(3 , 1.38%): 35, 148, 210 N-glycol(0): regulatory proteins |
| Rv1448 Transaldolase , tal, , protein Length = 373 amino acids | O-glycol(12 , 3.21%): 13, 17, 19, 94, 101, 123, 129, 166, 196, 200, 254, 266 N-glycol(1 , 0.27%): 67 intermediary metabolism and respiration |
| Rv0571c REVERSED Putative phosphoribosyl transferase, Rv0571c, protein Length = 443 amino acids | O-glycol(13 , 2.93%): 22, 135, 262, 264, 279, 316, 325, 336, 337, 358, 369, 406, 442 N-glycol(2 , 0.45%): 194, 271 conserved hypotheticals |
| Rv0928 Phosphate-binding protein PstS 3 , pstS3, protein Length = 370 amino acids | O-glycol(36 , 9.73%):21, 30, 35, 36, 40, 54, 56, 57, 64, 86, 91, 103, 121, 141, 143, 161, 188, 193, 205, 216, 232, 238, 241,248, 256, 263, 272, 275, 279, 286, 325, 337, 338, 344, 363, 364 N-glycol(6 , 1.62%): 28, 80, 157, 164, 206, 245 cell wall and cell processes |
| RV0120c Elongation factor G-like protein , GN=Rv0120c, protein Length = 714 amino acids | O-glycol(34 , 4.76%): 8, 16, 32, 37, 51, 55, 75, 95, 137, 179, 209, 269, 270, 282, 287, 338, 345, 350, 354, 355, 362, 407, 486, 494, 497, 522, 542, 595, 598,646, 657, 690, 694, 704 N-glycol(2 , 0.28%): 6, 18 information pathways |
| Rv3215 Putative isochorismate synthase, MenF , protein Length = 372 amino acids | O-glycol(17 , 4.57%): 2, 22, 44, 46, 159, 165, 173, 181, 213, 249, 257, 267, 269, 323, 330, 358, 366N-glycol(2 , 0.54%): 216, 265 intermediary metabolism and respiration |
| Rv1484Enoyl-[acyl-carrier-protein] reductase [NADH] , inhA, protein Length = 269 amino acids | O-glycol(12 , 4.46%): 13, 17, 19, 94, 101, 123, 129, 166, 196, 200, 254, 266 N-glycol(1 , 0.37%): 67 lipid metabolism |
| Rv2195 Cytochrome bc1 complex Rieske iron-sulfur subunit , qcrA, protein Length = 429 amino acids | O-glycol(22 , 5.13%): 19, 43, 58, 95, 149, 176, 195, 206, 248, 251, 258, 267, 300, 340, 350, 354, 360, 377, 402, 427, 428, 429 N-glycol(2 , 0.47%): 187, 412 intermediary metabolism and respiration |
| Rv3292 Uncharacterized protein Rv3292, protein Length = 415 amino acids | O-glycol(17 , 4.1%): 21, 62, 102, 108, 129, 193, 209, 246, 250, 268, 286, 305, 331, 350, 362, 369, 375N-glycol(0): conserved hypotheticals |
| Rv3283 Putative thiosulfate sulfurtransferase, SseA , protein Length = 297 amino acids | O-glycol(5 , 1.68%): 12, 22, 27, 144, 206 N-glycol(2, 0.67%):123, 269 intermediary metabolism and respiration |
| Rv2140c protein Rv2140c, protein Length = 176 amino acids | O-glycol(10 , 5.68%): 3, 4, 21, 23, 32, 36, 47, 53, 75, 151, N-glycol(1 , 0.57%): 88 conserved hypotheticals |
| Rv3726 Possible dehydrogenase , Rv3726, protein Length = 397 amino acids | O-glycol(14 , 3.53%): 18, 56, 93, 114, 191, 198, 212, 220, 273, 295, 300, 356, 358, 390 N-glycol(1 , 0.25%): 137 intermediary metabolism and respiration |
| Rv3141 Probable NADPH quinone oxidoreductase, FadB4, protein Length = 323 amino acids | O-glycol(14 , 4.33%): 25, 69, 74, 78, 89, 153, 154, 171, 172, 224, 277, 285, 307, 312 N-glycol(3 , 0.92%): 109, 253, 310 lipid metabolism |
| Rv3864 ESX-1 secretion-associated protein, EspE, protein Length = 402 amino acids | O-glycol(24 , 5.97%): 3, 5, 10, 11, 35, 37, 44, 54, 90, 139, 157, 227, 259, 304, 323, 325, 340, 344, 347, 354, 355, 390, 393, 400 N-glycol(3 , 0.75%): 13, 87, 160 cell wall and cell processes |
| Rv2140c protein Rv2140c,GN=Rv2140c, protein Length = 176 amino acids | O-glycol(10 , 5.68%): 3, 4, 21, 23, 32, 36, 47, 53, 75, 151 N-glycol(1 , 0.57%): 88 conserved hypotheticals |
| Rv3303c NAD(P)H dehydrogenase (quinone) , lpdA, protein Length = 493 amino acids | O-glycol(29 , 5.9%): 3, 24, 29, 51, 57, 58, 98, 107, 122, 139, 141, 152, 250, 252, 277, 282, 305, 319, 326, 345, 355, 370, 376, 409, 410, 424, 452, 456, 492 N-glycol(1 , 0.2%):280 intermediary metabolism and respiration |
| Rv1454c Probable quinone reductase Qor (NADPH:quinoneructase), protein Length = 328 amino acids | O-glycol(16 , 4.88%): 48, 61, 75, 82, 89, 116, 171, 224, 231, 245, 261, 275, 280, 292, 296, 323, N-glycol(1 , 0.3%): 93 intermediary metabolism and respiration |
| Rv0097 Putative dioxygenase Rv0097, protein Length = 289 amino acids | O-glycol(6 , 2.11%): 82, 92, 178, 193, 242, 266 N-glycol(1, 0.35%): 176 intermediary metabolism and respiration |
| Rv3559c REVERSED Probable oxidoreductase , Rv3559c , protein Length = 384 amino acids | O-glycol(22 , 5.73%): 54, 75, 77, 97, 102, 104, 186, 234, 260, 267, 272, 273, 277, 292, 294, 309, 348, 349, 354, 356, 364, 366 N-glycol(1 , 0.26%): 232intermediary metabolism and respiration |
| Rv3099c Conserved protein GN=Rv3099c, protein Length = 283 amino acids | O-glycol(21 , 7.42%): 3, 9, 43, 55, 60, 74, 101, 113, 132, 133, 134, 136, 140, 167, 168, 177, 187, 215, 219, 256, 260, N-glycol(0): conserved hypotheticals |
| Rv3532 Uncharacterized PPE family protein PPE61 , Rv3532, protein Length = 406 amino acids | O-glycol(43 , 10.59%): 14, 19, 25, 37, 53, 63, 64, 79, 81, 88, 90, 93, 97, 125, 132, 140, 159, 160, 165, 170, 171, 176, 178, 185, 239, 257, 260, 266, 269, 286, 308, 312, 314, 325,333, 338, 349, 350, 360, 361, 381, 400, 404 N-glycol(3 , 0.74%): 13, 126, 262 PE/PPE , PPE61 |
| Rv0752c Probable acyl-CoA dehydrogenase FadE9, proteinLength = 390 amino acids | O-glycol(13 or 3.33%): 14, 82, 98, 130, 136, 144, 160, 183, 191, 195, 241, 254, 389 N-glycol(1 , 0.256%): 244 lipid metabolism |
| Rv1977 Conserved protein, Rv1977, proteinLength = 348 amino acids | O-glycol(19 , 5.45%): 2, 4, 8, 16, 27, 61, 97, 116, 140, 158, 186, 232, 253, 261, 266, 330, 335, 341, 347 N-glycol(1 , 0.287%): 331 conserved hypotheticals |
| Rv0861c DNA helicase ercc3, proteinLength = 542 amino acids | O-glycol(14 , 2.58%): 103, 185, 191, 216, 217, 239, 259, 261, 282, 384, 450, 460, 473, 503N-glycol(1 , 0.18%): 458 information pathways |
| Rv2145c Cell wall synthesis protein Wag31, proteinLength =260 amino acids | O-glycol(10 , 3.48%): 69, 73, 92, 113, 167, 169, 192, 197, 239, 245 N-glycol(2 , 0.77%): 135, 260 cell wall and cell processes |
| Rv1658Argininosuccinate synthase , argG , protein Length = 398 amino acids | O-glycol(15 , 3.76%): 2, 15, 20, 91, 94, 119, 172, 175, 229, 236, 331, 336, 355, 372, 376 N-glycol(2 , 0.502): 79, 245 intermediary metabolism and respiration |
| Rv3676CRP-like cAMP-activated global transcriptional regulator crpprotein Length = 224 amino acids | O-glycol(14 , 6.25%):52, 75, 82, 90, 91, 92, 94, 97, 103, 144, 163, 172, 210, 216 N-glycol(0): regulatory proteins |
| Rv2296 Haloalkane dehalogenase 1 , dhmA1 proteinLength =300 amino acids | O-glycol(7 , 2.33%): 64, 70, 157, 218, 219, 295, 300 N-glycol(1, 0.33%): 147 intermediary metabolism and respiration |
| Rv0414Thiamine-phosphate synthase , thiE, Length = 222 amino acids | O-glycol(5 , 2.25%): 115, 120, 203, 216, 219 N-glycol(0): intermediary metabolism and respiration |
| Rv1533monooxygenase Rv1533, Rv1533, proteinLength = 375 amino acids | O-glycol(17 , 4.53%): 29, 43, 111, 116, 117, 118, 125, 172, 198, 269, 275, 279, 291, 299, 309, 313, 365 N-glycol(1 , 0.26%): 30 conserved hypotheticals |
| RV0873Probable acyl-CoA dehydrogenase FadE10, proteinLength = 650 amino acids | O-glycol(32 , 4.92%): 70, 102, 110, 122, 133, 137, 149, 166, 184, 185, 222, 230, 238, 286, 293, 331, 339, 372, 394, 424, 465, 502, 520, 542, 553, 590, 618, 624, 630, 643, 645, 646N-glycol(6 , 0.92%): 108, 288, 359, 418, 509, 587 lipid metabolism |
| Rv2211c Aminomethyltransferase, Rv2211c, proteinLength = 379 amino acids | O-glycol(23 , 6.06%): 14, 34, 52, 59, 67, 85, 88, 108, 134, 150, 152, 157, 168, 192, 204, 217, 230, 263, 326, 327, 331, 333, 345 N-glycol( 3, 0.79% , ): 55, 84, 130 intermediary metabolism and respiration |
| Rv2993c Possible 2-hydroxyhepta-2,4-diene-1,7-dioate isomerase , Rv2993c, proteinLength = 239 amino acids | O-glycol(6 , 2.5%): 15, 20, 74, 83, 176, 191 N-glycol(2 , 0.83%): 81, 118 intermediary metabolism and respiration |
| Rv2006Trehalose-phosphate phosphatase, otsB , proteinLength = 1327 amino acids | O-glycol(57 , 4,29%): 66, 96, 122, 129, 175, 192, 211, 255, 261, 303, 325, 357, 365, 428, 429, 502, 519, 563, 580, 657, 658, 681, 685, 692, 715, 725, 732, 733, 735, 745, 764, 774, 792, 803, 858, 861, 933, 935, 938, 950, 954, 1009, 1026, 1099, 1135, 1173, 1186, 1190, 1193, 1207, 1228, 1229, 1241, 1262, 1288, 1291, 1295, 1298 N-glycol(13 , 0.97%): 8, 121, 199, 334, 558, 600, 683, 947, 1031, 1137, 1138, 1205, 1208 virulence, detoxification, adaptation |
| Rv3528c Uncharacterized protein, Rv3528c proteinLength = 237 amino acids | O-glycol(6 , 2,53%): 26, 32, 76, 113, 141, 142 N-glycol(0): conserved hypotheticals |
| Rv1655Acetylornithine aminotransferase argD proteinLength = 400 amino acids | O-glycol (20 , 5%):2, 5, 7, 8, 10, 74, 75, 80, 105, 124, 143, 150, 228, 233, 275, 281, 282. 315, 343, 353 N-glycol(2 , 0.5%): 22, 117 intermediary metabolism and respiration |
| Rv3418c 10 kDa chaperonin , groS , protein Length = | O-glycol(6): 22, 24, 26, 33, 90, 99 N-glycol(1): 5 virulence, detoxification, adaptation |
| Rv3881c ESX-1 secretion-associated protein EspB , proteinLength = 460 amino acids | O-glycol(23 , 5%): 8, 52, 104, 112, 119, 182, 187, 241, 304, 307, 310, 313, 324, 326, 335, 341, 350, 359, 370, 375, 426, 429, 459 N-glycol(2 , 0.43%): 94, 129 cell wall and cell processes |
| Rv2677cCoproporphyrinogen III oxidase , hemY protein Length = 452 amino acids | O-glycol(23 , 5.08%): 14, 79, 85, 86, 94, 105, 111, 112, 137, 142, 168, 174, 186, 196, 210, 255, 293, 296, 313, 329, 347, 358, 421 N-glycol(0): intermediary metabolism and respiration |
| Rv1202Putative succinyl-diaminopimelatedesuccinylase , DapEproteinLength = 354 amino acids | O-glycol(12 , 3.38%): 41, 98, 129, 146, 156, 173, 181, 184, 221, 284, 306, 309 N-glycol(3 , 0.84%): 59, 243, 340 intermediary metabolism and respiration |
| Rv2560Uncharacterized, Probable proline and glycine rich transmembrane protein, Rv2560proteinLength = 325 amino acids | O-glycol(24 , 7.38%): 2, 23, 44, 76, 77, 80, 86, 98, 125, 130, 135, 136, 139, 140, 142, 146, 203, 240, 246, 250, 256, 261, 264, 302 N-glycol(3 , 0.92%): 95, 134, 257 cell wall and cell processes |
| Rv1077Putative cystathionine beta-synthase , cbs, proteinLength =464 amino acids | O-glycol(25 , 5.38%): 8, 21, 29, 66, 71, 72, 75, 160, 161, 169, 174, 182, 187, 197, 211, 214, 251, 313, 338, 352, 366, 387, 407, 424, 458 N-glycol(2 , 0.43%): 38, 74 intermediary metabolism and respiration |
| Rv1894c NMO domain-containing protein , Rv1894cprotein Length = 376 amino acids | O-glycol(20 , 5.31%): 3, 29, 43, 78, 91, 119, 144, 164, 174, 205, 229, 241, 246, 248, 259, 271, 277, 281, 301, 314 N-glycol(2 , 0.53%): 148, 371 conserved hypotheticals |
| Rv0734Methionine aminopeptidase 2, mapALength = 266 amino acids | O-glycol(14 , 5.26%): 17, 46, 47, 62, 67, 77, 92, 121, 128, 135, 138, 176, 214, 256 N-glycol(0): intermediary metabolism and respiration |
| Rv1901CinA-like protein OS=Mycobacterium tuberculosis, cinALength = 430 amino acids | O-glycol(27 , 6.27%): 4, 18, 70, 71, 77, 125,140, 148, 175, 180, 206, 211, 267, 293, 307, 308, 319, 341, 360, 362, 365, 371, 381, 411, 422, 424, 429 N-glycol(1 0.23%): 403 virulence, detoxification, adaptation |
| Rv1692 D,L-glycerol 3-phosphate phosphatase , Rv1692Length = 353 amino acids | O-glycol(21 , 5.94%): 3, 26, 34, 38, 64, 72, 73, 76, 126, 128, 129, 168, 175, 177, 225, 234, 256, 261, 339, 348, 349 N-glycol(3, 0.84%): 46, 101, 166 intermediary metabolism and respiration |
| Rv2065Precorrin-8X methylmutase, cobHLength = 208 amino acids | O-glycol(11, 5.28%): 76, 84, 86, 112, 113, 115, 138, 167, 182, 193, 205 N-glycol(1 , 0.48%): 201 intermediary metabolism and respiration |
| Rv1996Universal stress protein Rv1996proteinLength =317 amino acids | O-glycol(11, 3.47%): 94, 95, 96, 114, 119, 142, 145, 182, 278, 293, 299 N-glycol(2 , 0.63%): 117, 298 virulence, detoxification, adaptation |
| Rv1633 UvrABC system protein B uvrBproteinLength =698 amino acids | O-glycol(25, 3.58%): 11, 43, 48, 50, 51, 130, 155, 194, 201, 241, 248, 291, 371, 403, 533, 535, 539, 548, 561, 608, 616, 621, 635, 644, 645 N-glycol(4 , 0.57%): 68, 309, 346, 619 information pathways |
| Rv0015cSerine/threonine-protein kinase PknA proteinLength =431 amino acids | O-glycol(34 , 7.9%): 2, 21, 49, 64, 82, 90, 129, 172, 180, 198, 212, 224, 263, 284, 286,299, 301, 302, 309, 313, 316, 319, 323, 334, 335, 373, 375, 377, 378, 385, 390, 393, 407, 417 N-glycol(3 , 0.7%): 69, 104, 397 Regulatory proteins |
| Rv1629DNA polymerase I , polAproteinLength =904 amino acids | O-glycol(36 , 3.98%): 3, 4, 9, 46, 53, 66, 75, 146, 214, 224, 249, 257, 379, 417, 418, 420, 452, 497, 498, 517, 557, 583, 590, 631, 645, 657, 682, 714, 736, 744, 749, 791, 792, 805, 826, 898 N-glycol(5 , 0.55%): 47, 90, 231, 649, 820 information pathways |
| Rv1502Uncharacterized protein , Rv1502, proteinLength =299 amino acids | O-glycol(18 , 6.02%): 13, 21, 49, 50, 53, 90, 110, 116, 122, 132, 165, 173, 201, 239, 260, 263, 283, 290 N-glycol(1 , 0.33%): 47 unknown |
| Rv0583c Probable conserved lipoprotein, LpqN , proteinLength =228 amino acids | O-glycol(27 , 11.84%): 5, 10, 14, 28, 31, 32, 33, 35, 36, 38, 40, 41, 42,43, 44, 45, 46, 47, 49, 50, 51, 154, 158, 166, 173, 192, 225 N-glycol(2 , 0.88%):58, 202cell wall and cell processes |
| Rv1284 Beta-carbonic anhydrase 1 , mtcA1protein Length = 163amino acids | O-glycol(7 , 4,29%): 27, 64, 70, 74, 119, 145, 162 N-glycol(2 , 1.22%): 137, 159 Intermediary metabolism and respiration |
| Rv0838 D-alanyl-D-alanine dipeptidase, lpqR, protein Length = 256amino acids | O-glycol(20 , 7.81%): 27, 32, 36, 38, 39, 47, 49, 51, 58, 84, 89, 158, 164, 168, 171, 178, 206, 210, 230, 233 N-glycol(3 , 1.17%): 46, 86, 216 cell wall and cell processes |
| Rv0183Monoacylglycerol lipase , Rv0183protein Length = 256amino acids | O-glycol(6 , 2.15%): 3, 27, 71, 82, 110, 243 N-glycol(1 , 0.36%): 9 Intermediary metabolism and respiration |
| Rv0593Possible Mce-family lipoprotein LprLprotein Length = 402aminoacids | O-glycol(26 , 6.47%): 6, 9, 24, 30, 44, 83, 99, 122, 128, 129, 145, 151, 154, 161, 178, 230, 244, 262, 272, 277, 294, 341, 357, 359, 362, 378 N-glycol(5 , 1.24%): 82, 159, 194, 242, 339, |
| Rv2557Uncharacterized protein, Rv2557protein Length = 224aminoacids | O-glycol(14 , 6.25%): 2, 12, 23, 25, 35, 72, 76, 88, 118, 168, 173, 174, 179, 190 N-glycol(0): Conserved hypotheticals |
| RV1418 Putative lipoprotein , lprH, protein Length = 228 aminoacids | O-glycol(15 , 6.58%): 43, 44, 49, 72, 79, 82, 83, 90, 98, 116, 156, 161, 187, 203, 226 N-glycol(3 , 1.32%): 114, 133, 183 cell wall and cell processes |
| Rv2799 Probable membrane protein , Rv2799protein Length = 209aminoacids | O-glycol(17 , 8.13%): 17, 37, 48, 57, 63, 64, 65, 68, 73, 130, 139, 154, 162, 163, 171, 178, 182 N-glycol(0): cell wall and cell processes |
| Rv1270cLipoprotein LprA, LprA , protein Length = 244aminoacids | O-glycol(25 , 10.25%): 13, 26, 35, 37, 40, 42, 61, 94, 96, 98, 105, 135, 164, 172, 176, 179, 180, 186, 194, 202, 208, 211, 212, 222, 225 N-glycol(4 , 1.64%): 43, 103, 138, 178 cell wall and cell processes |
| Rv0222Probable enoyl-CoA hydratase EchA1, protein Length = 262aminoacids | O-glycol(27 , 10.31%): 2, 41, 63, 68, 130, 166, 189, 202, 217, 219, 223, 241 N-glycol(2 , 0.76%): 37, 242 lipid Metabolism |
| Rv1388Putative integration host factor MihF , MihF, protein Length = 190 aminoacids | O-glycol(3 , 1.58%): 21, 143, 189 N-glycol(1 , 0.53%): 123 information pathways |
| Rv0934 Periplasmic phosphate-binding lipoprotein PstS1 (PBP-1) pstS1, protein Length = 374 aminoacids | O-glycol(34 , 9.09%): 26, 30, 32, 35, 41, 44, 45, 48, 49, 52, 56, 59, 80, 82, 86, 88, 128, 186, 192, 210, 215, 216, 236, 241, 269, 280, 290, 292, 299, 328, 341, 347, 373, 374 N-glycol(3 , 0.8%): 146, 229, 268 cell wall and cell processes |
| Rv0248cProbable succinate dehydrogenase [iron-sulfur subunit] Rv0248c, protein Length = 646 aminoacids | O-glycol(27 , 4.18%): 40, 47, 69, 74, 117, 150, 194, 220, 222, 239, 251, 258, 281, 312, 331, 339, 363, 400, 421, 430, 451, 457, 470, 594, 597, 599, 602 N-glycol(5 , 0.77%): 221, 280, 504, 603, 607 Intermediary metabolism and respiration |
